# Influence of climatic factors on *Ixodes ricinus* nymph abundance and phenology over a long-term monthly observation in Switzerland (2000–2014)

**DOI:** 10.1186/s13071-018-2876-7

**Published:** 2018-05-08

**Authors:** Gaël Hauser, Olivier Rais, Francisca Morán Cadenas, Yves Gonseth, Mahmoud Bouzelboudjen, Lise Gern

**Affiliations:** 1 Institute of Biology, University of Neuchâtel, Emile Argand 11, 2000 Neuchâtel, Switzerland; 2 Centre Suisse de Cartographie de la Faune, Avenue de Bellevaux 51, 2000 Neuchâtel, Switzerland; 3 Service informatique et télématique, University of Neuchâtel, Emile Argand 11, 2000 Neuchâtel, Switzerland

**Keywords:** *Ixodes ricinus*, Ticks, Population density, Climatic factors, Long-term observation, Phenology

## Abstract

**Background:**

One of the major public health challenges in the field of communicable diseases consists of being able to predict where and when a population is at risk of being infected by a pathogen. In the case of vector-borne diseases, such predictions often require strong ecological knowledge of the vector life-cycle and the environmental conditions promoting or preventing its establishment and maintenance. In this study, we analyse how climatic factors influence the abundance and phenology of the Lyme borreliosis vector *Ixodes ricinus* in a Swiss temperate forest*,* based on a long-term monthly observation over a period of 15 years (2000 and 2014).

**Results:**

Our results show that questing nymph density significantly decreased during the study period in the sampling area. Although the analyses of climatic variables point out the relative importance of air temperature, relative humidity and saturation deficit on nymph questing activity, the global trends followed by these variables over the study period failed to fully explain the observed decline. However, nymph phenology was additionally explained by the presence of climatic thresholds that limit the questing behaviours of ticks. Most notably, we found that the presumed upper threshold of air saturation deficit, which strongly limits the increase of questing nymph density and is typically reached in the middle of spring, was reached significantly earlier and earlier over years.

**Conclusions:**

In addition to phenology *per se*, the use of climatic thresholds may help to predict the presence and abundance of questing ticks in Lyme borreliosis endemic areas. Tick sensitivity to temperature or saturation deficit thresholds also suggests that extreme climatic events more than global trends may affect tick population dynamics. These two points may be of high importance in epidemiological short-term as well as long-term predictions. However, the highly unexplained variability in nymph density underlines the need for further studies that include other factors such as tick host abundance or tick microhabitats, two potentially influent factors that were not assessed in the present study.

**Electronic supplementary material:**

The online version of this article (10.1186/s13071-018-2876-7) contains supplementary material, which is available to authorized users.

## Background

In Europe, ticks, particularly *Ixodes ricinus*, are important vectors for etiological agents of zoonotic diseases (among others, tick-borne encephalitis virus and Lyme borreliosis spirochetes) [1]. Tick-borne diseases are of increasing significance for public health in North America [2], and in Europe [3]. *Ixodes ricinus* has a very wide geographical distribution throughout Europe [4] and is also reported in North Africa (Tunisia, Algeria and Morocco) [5].

*Ixodes ricinus* displays a seasonal activity generally from February to November [6]. This tick species is very generalist, feeding on numerous mammalian, avian and reptilian species and goes through three active life stages: larva, nymph, and adult. It is mainly observed in the leaf litter and the low strata vegetation of deciduous woodlands and mixed forests [6].

Risk of infection by vector-borne agents depends on the frequency of contacts between humans and vectors that are infected, which is influenced by biotic and abiotic factors [3]. The host-finding behaviour of *I. ricinus* ticks is formed by successive steps; one of these, questing on vegetation, is crucial. However, during questing, ticks experience various climatic conditions that may be deleterious for them [6]. *Ixodes ricinus* is very sensitive to temperature and humidity compared to other ticks [7, 8]. Combined with temperature, humidity is an important limiting factor in *I. ricinus* survival and activity since all developmental stages do not resist to desiccation [9–11]. In fact, relative humidity (RH) needs to be above 70–80% in order to allow questing tick activity and survival [9]. Therefore, ticks regularly interrupt questing on vegetation and move down to moist surroundings to rehydrate [10, 12]. Saturation deficit (SD), a measure of the drying power of the atmosphere depending on both temperature and RH [13], increases tick walking distance [14], limits duration of questing [12, 15] and survival of *I. ricinus* in nature [15–20]. Herrmann & Gern [21] confirmed, in a laboratory study, that increasing relative humidity influenced positively *I. ricinus* survival whereas increasing temperature affected it negatively. As a combination of these two factors, SD had a stronger impact on *I. ricinus* than temperature or RH alone. Hence, survival rate was inversely proportional to SD, i.e. more ticks died as SD increased.

In this study, we analysed the variations in the seasonal questing activity and abundance of *I. ricinus* nymphs over a period of 15 years in a forest in Switzerland in relation with climatic factors. Several previous studies showed that weather conditions such as temperature [22–25], saturation deficit [17, 20, 24, 26, 27] or precipitation [3] may impact *Ixodes* tick density and phenology. A recent study identified several important areas of uncertainty concerning Lyme borreliosis ecology, among which the influence of climate and climate change on tick abundance and phenology was mentioned [27]. The present analysis of a long-term set - over fifteen years - of monthly observations of *I. ricinus* nymph abundance and micro- and macroclimate at one site [17] may be considered as a coherent case study to test and discuss the main predictions around the still exploratory question of tick density and population variation in a changing climate context. We focused on the nymphal stage, as nymphs are mainly concerned in the transmission of Lyme borreliosis agents or tick-borne encephalitis (TBE) virus in Europe.

## Methods

### Tick sampling

*Ixodes ricinus* ticks were collected once a month in a mixed forest dominated by deciduous trees (*Quercus* spp., *Fagus* spp., *Fraxinus* spp. and *Prunus* spp.) (Bois de l’Hôpital) on the south-east-facing slope of the Chaumont Mountain (47°01'N, 06°56'E, Neuchâtel, Switzerland, Additional file 1: Figure S1) between years 2000 and 2014. During this period no change in forestry or landscape occurred in the studied area. Rainy and windy days were excluded from sampling. One person (OR) sampled the ticks over the whole study period by flagging the low vegetation using a 1 m^2^ cotton cloth along a 1049 m long circular footpath, representing a total sampled surface of 1049 m^2^. Ticks were counted and collected from the flag every 17.5 m. The altitude of the sampling zone varies from 520 m to 585 m above sea level. Ticks were identified to species, stage and sex for the adults, and the questing tick density was expressed as the number of individuals collected per 100 m^2^. We focused our study on the nymphal stage because it is the most frequent tick stage collected in the area (larvae were scarce, probably questing too close to the ground to be collected by the flag). During the first study years (2000–2005) tick sampling was excluded when snow covered the vegetation: missing samplings are December 2000 and 2002, January 2003, January and December 2004, January, February, November and December 2005. After 2005, one last sampling, December 2012, was missing. Thus, over the 15 years, 170 monthly samplings were performed. Among them, three were done over two different days. In analyses exploring the link between nymph density and weather conditions, data of these three samplings were considered as independent data points (therefore *n* = 173), as climatic conditions - and so nymph questing behaviour - might have differed from one day to the next.

### Climatic data

Temperature and relative humidity were measured at each sampling directly at the collection site (550 m above sea level) using a thermo-hygrometer placed 60 cm above ground (model 615, Testo SA, Lonay, Switzerland). From these data, saturation deficit (SD), which integrates temperature and relative humidity to derive a measure of the drying power of the atmosphere, was calculated according to Randolph & Storey [15]:

$$ \mathrm{SD}=\left(1\hbox{-} \mathrm{RH}/100\right)\times 4.9463\times {\mathrm{EXP}}^{\left(0.0621\times \mathrm{T}\right)} $$

where SD is the air saturation deficit expressed in mm Hg, RH/100 is the air relative humidity in percent, and T is the measured air temperature in degree Celsius.

In addition, daily temperature (mean and maximum), daily mean of relative humidity and daily sum of precipitations were obtained from Climap-net database of the Federal Office for Meteorology and Climatology (MeteoSwiss). The selected measuring station is located in Neuchâtel (47°00'N, 6°57'E), at about 450 m from sampling site as the crow flies, and at 485 m of altitude, which is between 35 and 100 m lower than the area covered by the samplings. Daily air saturation deficit was calculated the same way as for field data but considering daily mean values for temperature and relative humidity. Climap-net data allowed us to take into account the weather conditions of the days preceding the nymph sampling to evaluate their impact on *I. ricinus* questing behaviour on five different time scales (daily mean and 5, 10, 17 and 30 days moving averages), as field data were only available for the day of sampling.

### Nymph density parameters

**Fig. 1:**
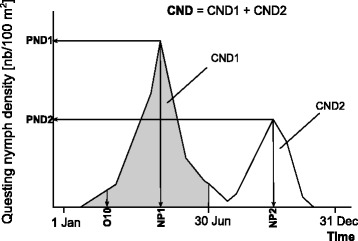
Schematic view of a density curve of questing nymphs over one year, modified from Perret et al. [15]. Shown are parameters used for analyses: cumulated nymph density (CND), calculated as the integral of nymph density curve over the year; peak nymph density (PND) represents the maximal nymph densities in spring (PND1) and autumn (PND2); nymph peak (NP1 and 2) are dates linked to PND1 and 2, respectively. O10 represents the first day of the year at which nymph density reaches at least 10% of maximal density (PND1). This date was interpolated from the 2 closest field samples

To assess for any long-term trend in nymph density, we estimated the number of nymphs/100 m^2^/year by integrating the area under the density curve for each year [cumulated nymph density (CND) [28], Fig. 1]. The CND value thus represents the total nymph density expected to be collected during an entire year if the sampling is done every day during that year. If we divide CND by 365, we would therefore obtain the daily mean of nymph density based on the density curve. As samplings during cold months were missing during year 2000 to 2005, CND of these years may be underestimated. To adjust, fictive dates where added to the database when we expected nymph density to be null, according to a discriminating temperature threshold that has been previously shown to limit nymph activity in the study site [17] (Additional file 1: Figure S2). More specifically, for each year that did not begin or end with a null sampling (0 nymph collected), the closest date below the temperature threshold (7 °C of the 5-day moving average of daily maximal temperature) was identified in the Climap-net database and added as an arbitrary null sampling. A total of 20 fictive samplings were added using this method. These 20 fictive samplings were used to calculate and graphically represent CND’s for each year as well as to determine the nymph activity onset date (O10) when relevant (see below) but these fictive samplings were not considered in any other analysis. Differentiate calculations were also made for cumulated questing nymph density during the first density peak in spring (CND1), and the second peak in autumn (CND2). These separated calculations were used to assess whether nymph activity during spring and autumnal periods changed in different ways. To differentiate the areas belonging to both peaks, each year was separated into two semesters with an arbitrary limit date fixed on 30th of June. The nymph activity onset date (O10) was also considered, it represents the first day of the year at which nymph density reaches 10% of the peak density of that year. This date was linearly interpolated from the two closest sampling dates. Additional descriptive parameters (mean, maximum and minimum density) are listed in Table 1 and main phenological parameters can be visualized in Fig. 1.

**Table 1:** Descriptive statistics for nymph density from 2000 to 2014. Yearly mean, maximum and minimum densities of field-collected questing nymphs during the study period

Year	Mean density	Maximum density	Minimum density	Cumulated density (CND)
2000	30.49	100.48	0.19	11,147.38
2001	38.60	146.71	0.10	14,697.43
2002	36.66	132.22	1.14	12,564.97
2003	36.03	163.11	0.10	13,612.82
2004	30.22	105.62	1.62	9295.09
2005	22.34	49.95	4.10	5649.52
2006	38.73	159.68	0	13,735.11
2007	17.04	51.48	0	6999.33
2008	35.47	106.39	0	12,769.21
2009	30.22	134.51	0	11,227.26
2010	20.36	69.32	0	6683.16
2011	32.69	97.09	0	10,830.31
2012	17.52	61.24	0.19	6457.33
2013	22.40	96.38	0	8221.73
2014	17.62	49.71	0	6385.29

*Notes*: Densities are indicated in number of questing nymphs per 100 m^2^. Cumulated nymph density (CND) is calculated as the area under the density curve over the years. For this latter measure only (CND), fictive null samplings were considered (see Methods for details)

### Statistical analyses

For each climatic factor (temperature, relative humidity, air SD and precipitations), daily mean and moving averages (MA) (5, 10, 17 or 30 days) were calculated to take into account different time periods potentially relevant in determining nymph activity [17].

In the first part of the analyses, we investigated the effects of the above listed climate variables on nymph questing activity. The response variable was the rounded value of nymph density (number of nymphs/100 m^2^) at each sampling date, and climatic variables were used as explanatory variables. As the rounded values of nymph density are similar to count data, we used generalized linear models (GLM) with negative binomial probability distribution (NBGLM). GLM with Poisson distribution of errors was tested first but showed high degree of overdispersion (overdispersion test from the AER library [29] in R). NBGLM was therefore more adequate [30].

Six different models were built according to the time scale of interest: (i) field measures; (ii) daily means (weather station); (iii) 5-days moving averages (MA); (iv) 10-days MA; (v) 17-days MA; and (vi) 30-days MA. Each model tested the effect of temperature, relative humidity and precipitations on the density of nymphs at the mentioned time scales. Saturation deficit was tested separately and in replacement of temperature and humidity, as it is highly collinear with these variables. To discriminate between weather effects and temporal pattern on nymph questing activity, a function of time (date) was added to the model as a predictor, as shown in Qviller et al. [31]. To reflect the non-linear and season-dependent shape of nymph phenology, we used the natural cubic spline function of the date and put it as predictor in each of the tested models [31]. For each model, a backward stepwise selection based on AIC was done to obtain an optimised model for each time scale [instantaneous (field data), daily mean, 5, 10, 17 and 30 MA]. Finally, among the 6 final models (each representing the optimised model at a given time scale) we used AIC values to determine which model - and therefore which time scale - had the highest explanatory power for nymph density. As field measurements were lacking in 19 of the 173 samplings, these samplings were removed from the dataset before the analysis (thus, *n* = 154 instead of *n* = 173). In fact, models must be built from the same dataset and contain the same number of data to properly compare AIC. Model comparisons based on likelihood ratio tests (LR) were additionally performed. Precipitation variable was not tested in the field model or daily mean model, as nymph samplings were done only by dry weather, which introduces a systematic bias for these two time scales.

Second part of the analyses focused on yearly data points (i.e. CND, peak density or nymph onset date over years). Linear regressions were used to statistically assess long-term trends and to evaluate changes in phenology over years using yearly data points. Although CND can be viewed as the annual sum of daily samplings (and thus the sum of count data), doing this calculation avoids our data points to be along the bound of zero, and their distribution was not inflated toward low numbers. Moreover, these analyses were not aimed to predict trends out of our data limits but to focus on describing trends inside our data. Therefore, we considered linear models to be suited for these explorative analyses. For the latter models, normality of residuals was checked using Shapiro-Wilk tests. For all tests, an alpha error of 0.05 was considered as the threshold to define statistical significance.

For long-term trends of temperature, relative humidity and saturation deficit, we used generalized least squares (GLS) [32] to account for autocorrelation of model residuals, as proposed in [33]. Daily mean values were considered as the response variable, and day as the time explanatory variable. For precipitations, as model residuals did not show obvious autocorrelation, we used a generalized linear model (GLM) with gamma distribution to assess for any trend over time.

The data were analysed using R, version 3.2.5 [34], and JMP®, Version 12.1 SAS Institute Inc., Cary, NC, 1989–2017.

## Results

### Questing nymph density

A total of 8722 *I. ricinus* larvae, 50,201 nymphs and 8574 adults were collected during the study period*.* On average, 28 ± 36 (mean ± standard deviation, SD) nymphs/100 m^2^ were collected during one sampling. Questing nymph density showed variations among samplings, ranging from a minimum density value of null during cold months to a maximum density value of 163.11 nymphs/100 m^2^ for the highest peak density measured in spring 2003 (Table 1). Because of missing samplings during winter months in 2000, 2002–2005 and 2012, nymph samplings were all positive for these years, which contrasts with data from 2006 to 2014 (2012 excepted) showing null values due to unsuccessful samplings during winter months. There was one exception at the beginning of the study, during 2001 nymph samplings were done every month (*n* = 12) and all were positive, even in January and December.

Cumulated nymph density (CND) varied from 14,697.43 nymphs/100 m^2^ in 2001 to 5649.52 nymphs/100 m^2^ in 2005 (Table 1). Separate data for semesters one and two for each year are shown in Additional file 2: Table S1 and S2.

**Fig. 2:**
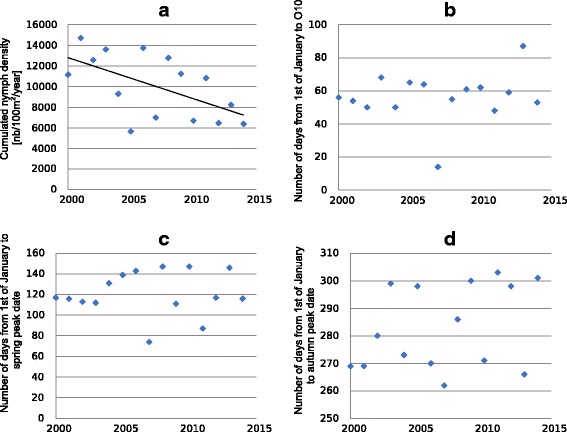
Questing density and phenology of nymphs during the study period (January 2000 to December 2014). **a** Cumulated nymph density. **b**-**d** Number of days from 1st January to onset dates (O10), to spring peak dates (NP1) and autumn peak dates (NP2), respectively

CND was found to decrease significantly over years (*F*_(1, 13)_ = 6.37, *r*^2^_Adj_ = 0.28, *P* = 0.02, Fig. 2a) with an annual drop of 3.01%. Cumulated nymph density during the spring peak (CND1) and the autumnal peak (CND2) followed the same negative trend with 3.04% annual decrease (*F*_(1, 13)_ = 4.23, *r*^2^_Adj_ = 0.19, *P* = 0.06), and 2.96% annual decrease (*F*_(1, 13)_ = 5.19, *r*^2^_Adj_ = 0.23, *P* = 0.04) for CND1 and CND2 respectively; Additional file 1: Figure S3 and S4).

**Fig. 3:**
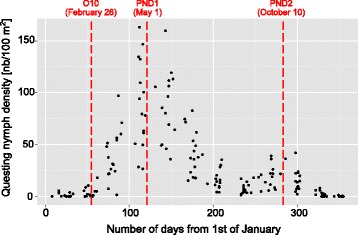
Superimposed nymph phenology from 2000 to 2014 field samples. Points represent nymph density for each sampling, in function of day and month of tick collection, regardless of year. The three vertical dotted lines represent mean dates for, from left to right, O10 (questing onset: 10% of peak density), PND1 (spring peak), and PND2 (autumnal peak)

We observed no evidence of change over the whole study regarding the period of questing nymph activity despite significant decrease in global nymph abundance. Indeed, the number of days from 1st of January to the first date when questing nymph density increased above 10% of the peak nymph density (also called here nymph questing onset date (O10, Fig. 2b), or the number of days from 1st of January to spring peak density date (NP1, Fig. 2c) or autumnal peak (NP2, Fig. 2d) did not show any significant trend over years despite important variations. On average, nymph questing onset date (O10) was reached on 26th of February, corresponding to 56.4 ± 15.2 days (mean ± SD) after January 1st. PND1 was reached after 121.1 ± 21.6 days (mean ± SD), corresponding to 1st of May, and PND2 was reached after 283 ± 15.3 days (mean ± SD, corresponding to 10th of October (Fig. 3).

### Climatic trends during the study period

Global variation of temperature, relative humidity and SD (Climap-net database) over the 15 years of study were analysed using generalized least squares (GLS). Daily mean temperature raised (*t* = 1.98, *df* = 5476, *P* = 0.048) with an average increase of 0.049 °C each year, implying an interpolated difference of +0.74 °C between 2000 and 2014. Daily mean values of relative humidity also increased during the same period (*t* = 4.64, *df* = 5476, *P* < 0.001) with an average increase of 0.18% each year, implying an interpolated difference of +2.7% between 2000 and 2014. No significant change was detected for daily mean of SD (*t* = -1.15, *df* = 5476, *P* = 0.25). Daily sums of precipitations were analysed using a generalized linear model with gamma distribution, but no trend was detected (*χ*^2^ = 0.07, *df* = 1, *P* = 0.79).

### Climatic variables and questing nymph density

Linear regressions between field and Climap-net database data showed that daily mean temperature and humidity fitted well with the corresponding field data (*F*_(1, 145)_ = 2185, *r*^2^_Adj_ = 0.95, *P* < 0.001 and *F*_(1, 139)_ = 167.3, *r*^2^_Adj_ = 0.57, *P* < 0.001, respectively; Additional file 1: Figure S5). The daily mean values differed from the field data by -4.4 °C for temperature and +2.8% for relative humidity at the intercept, but these differences were fairly constant across our data range due to slope values of 0.91 and 0.93, respectively (Additional file 1: Figure S5).

#### Temperature, relative humidity and precipitations

Number of degrees of freedom in the cubic spline function of the date (used to control for temporal pattern) was chosen after visual assessment of the resulting curve. We set the degrees of freedom to 6, as the curve convincingly represented the phenology curve and its dual peaks and showed no overfitting. If fitted alone as predictor, the date function had a highly significant effect on nymph density (*χ*^2^ = 485, *df* = 6, *P* < 0.001), and the AIC of the resulting model was 1095.6. A summary of the different NBGLM fitted is shown in Table 2. The lowest AIC was obtained from the model assessing the effect of field weather measurements on questing nymph density (AIC = 1047.5). AIC of the remaining models were found to increase along with the chosen time scale, which suggests that instantaneous weather measurements were the best set of predictors of questing nymph density, and 30 days moving averages were the worst set of predictors. Likelihood ratio tests confirmed that models using longer time scales (10, 17 and 30 days MA) were statistically different from the model with field measurements (*P* < 0.001). However, at shorter time scales (daily means and 5 days MA), model comparisons did not show statistically significant differences with the field model (*χ*^2^ = 2.16, *P* = 0.14 and *χ*^2^ = 3.6, *P* = 0.056, respectively). Finally, precipitations were not found to affect questing nymph density for any tested time scale. Variables included in each model and AIC are given in Table 2. Detailed output of the model with the lowest AIC (field measurements) is given in Table 3.

**Table 2:** Results of the six model selection procedures at different time scales

Climatic variable	Field measure	Daily mean (weather station)	5 day MA	10 day MA	17 day MA	30 day MA
Ns (date, *df* = 6)	×	×	×	×	×	×
T	×	×	×	×	×	×
T^2^	×	×	×	×	×	×
RH	×	×	×^*^	×^*^	×^*^	
RH^2^	×^*^			×^*^		
PR	NT	NT				
PR^2^	NT	NT				
AIC	1047.5	1047.7	1049.2	1054.9	1060.9	1085.0
∆AIC	0	0.2	1.7	7.4	13.4	37.5

*Notes*: Each column (starting from column 2) represents a model in which nymph density (nb/100 m^2^) was the response variable (*n* = 173), and the explanatory variables used in the full model are listed in the first column, i.e. temperature (T), relative humidity (RH), and precipitations (PR), with their respective quadratic terms. Crossed = term included in the model after backward stepwise selection based on Akaike Information Criterion (AIC). AIC and ∆AIC (difference with the model with the lowest AIC). Terms included (×) were statistically significant in their respective model output. Terms noted with an asterisk (×^*^) indicate a trend (*P* < 0.1) in the model output. Precipitations were not considered in the 2 first models (NT, not tested) due to biases induced by the arbitrary choice to sample nymph only during dry days. PR was not found to be significant in any of the tested models

**Table 3:** Output of the NBGLM explaining nymph density from field data

Coefficients	Estimate	SE	*Z*-value	*P*-value
Intercept	-3.99	0.88	-4.52	< 0.001
T	0.35	0.05	6.9	< 0.001
T^2^	-0.008	0.001	-5.82	< 0.001
RH	0.05	0.02	2.24	0.025
RH^2^	-0.0004	0.0002	-1.89	0.059
Ns (date, *df* = 6)1	3.45	0.51	6.71	< 0.001
Ns(date, *df *= 6)2	1.74	0.59	2.94	0.003
Ns(date, *df* = 6)3	-0.09	0.56	-0.16	0.87
Ns(date, *df* = 6)4	2.17	0.46	4.67	< 0.001
Ns(date, *df* = 6)5	2.28	0.93	2.44	0.015
Ns(date, *df* = 6)6	-2.58	0.59	-4.37	< 0.001

*Notes*: Ns is natural cubic spline and *df* gives the degrees of freedom chosen to calculate the spline. One estimate is given for each climatic variable and each section of the spline (given by the *df*). Null deviance = 832.7 on 153 *df*, residual deviance = 159.4 on 143 *df*, number of nymph sampling = 173, 19 weather data were missing

*Abbreviation*: *SE* standard error

#### Saturation deficit

The same procedure was followed to analyse the effect of SD that was put in replacement of both temperature and humidity in the models. SD or its quadratic term were not found to be significant at any time scale, but only showed a trend in the model using daily mean values (daily mean of SD: *χ*^2^ = 3.38, *df* = 1, *P* = 0.066, AIC = 1094.2). However, when the time predictor (cubic spline function of the date) was removed, SD and its quadratic terms were highly significant (*P* < 0.001) at all the tested time scales, although model AICs also largely increased (AIC = 1249.2 on average).

**Fig. 4:**
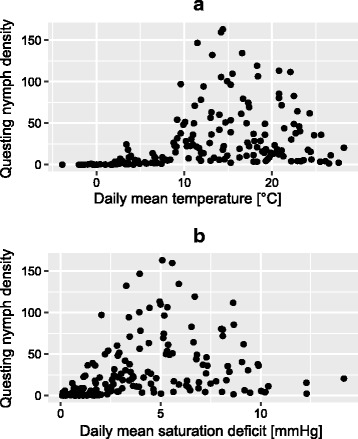
Correlations between temperature (**a**) or saturation deficit (**b**), and questing nymph density (nb/100m^2^)

As suggested by the importance of quadratic terms that showed negative estimates (Table 3) in the different NBGLMM, the shapes of the correlations between temperature and nymph density approached unimodal responses (Fig. 4). This remained true for temperature even after having controlled for temporal variation in the model. This implies that besides any temporal pattern of nymph activity, there are lower and upper thresholds below and above which the questing of nymphs may be strongly limited. In the case of SD, the shape is visually similar but the net effect could not be separated from the temporal pattern in our models, implying a strong link between phenology and SD. Existence and coherence of threshold values were assessed by visualisation of parallel plots showing questing nymph density and important climatic variables (Additional file 1: Figure S6) using 2- or 5-days moving averages, which were considered more insightful than daily means for this purpose because of the important variation at very short time scale. The relevance of the observed thresholds and their links to nymph density was assessed (see below).

### Questing nymph activity and climatic threshold values

Visual observation of nymph phenology during the whole period (2000 to 2014) and the parallel variation of climatic variables allowed us to point out the importance of temperature and SD in determining the general shape of density curves, which is likely sensitive to threshold values, as discussed above. Precipitation appeared highly variable and failed to convincingly explain the shape of questing tick density curves.

#### Temperature

A limit of 7 °C of the 5-day moving average of daily maximal temperature - a limit that was also used to add arbitrary null samplings (see Methods) was applied to discriminate between days with and without tick questing activity according to Perret et al. [17] (Additional file 1: Figure S2).

**Fig. 5:**
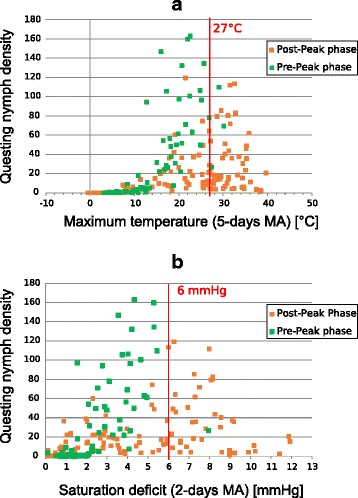
Correlation between maximal temperature (5-days moving average, **a**) or 2-days moving average of saturation deficit (**b**) and questing nymph density. Arbitrary thresholds are shown in red and indicate limits above which nymphs are scarcely found to be in their rising phase. Green squares represent the samplings done in the rising (pre-peak) phase (from 1st of January to PND1), orange squares represent the rest of the samplings (post-peak phase)

Based on Fig. 5a and Additional file 1: Figure S6, we also chose the value of 27 °C (5-days moving average of maximum temperature) as an upper threshold above which nymph density was mostly observed to decrease, i.e. samplings undertaken after the yearly peak density of nymphs (PND1). Figure 5a allows this visual observation by discriminating between samplings done before (pre-peak phase) and after PND1 (post-peak phase). The number of hot days during which the 5-day moving average of maximum temperature exceeded this defined upper limit of 27 °C showed a trend for a negative correlation with autumnal nymph density CND2 (*F*_(1, 13)_ = 142.4, *r*^2^_Adj_ = 0.15, *P* = 0.087), longer warm periods affecting negatively questing nymph density during autumnal peak. However, the number of these warm days did not decrease or increase significantly over years (Additional file 1: Figure S7; *F*_(1, 13)_ = 2.4, *r*^2^_Adj_ = 0.09, *P* = 0.15).

We further observed that average temperature during late winter, i.e. during January and February together, was significantly correlated with the nymph onset date O10 (*F*_(1, 13)_ = 7.33, *r*^2^_Adj_ = 0.31, *P* = 0.018), with colder winters delaying questing nymph activity onset. However, the date of this onset (O10) was not correlated with cumulated nymph density during the first semester CND1 (*F*_(1, 13)_ = 0.2, *r*^2^_Adj_ = -0.06, *P* = 0.66), and did not show any trend over years (*F*_(1, 13)_ = 0.38, *r*^2^_Adj_ = -0.05, *P* = 0.55, Fig. 2b).

#### Saturation deficit

Similarly to maximum temperature, a threshold of SD was used to discriminate between pre-peak period when questing density of nymphs is increasing before the spring peak (from 1st of January to PND1) and post-peak period (from PND1 to 31st of December). We considered the value of 6 mmHg of the 2-days moving average of SD as a limit above which questing nymph density values corresponded to post-peak samplings (except for 1 sampling, see Fig. 5b). On average, this threshold was reached after 111 ± 15 days (mean ± SD) during the studied period, that is around 22th of April.

**Fig. 6:**
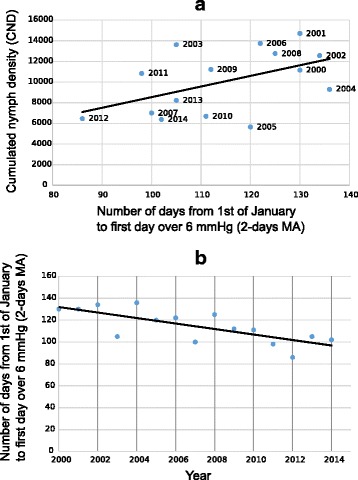
**a** Correlation between number of days from 1st January to the first date over 6mmHg (2-days moving average of saturation deficit), and yearly cumulated nymph density (CND). **b** Number of days from 1st of January to the first date over 6mmHg (2-days moving average of saturation deficit), from 2000 to 2014

The number of days from 1st of January to the first date when the SD value was exceeding 6 mmHg showed a trend for a positive correlation with yearly cumulated nymph density (CND) (*F*_(1, 13)_ = 4.28, *r*^2^_Adj_ = 0.19, *P* = 0.059), a longer period below this threshold value corresponding to a higher nymph density value (Fig. 6a), suggesting a causal link between this SD value and nymph activity. Moreover, this threshold was reached each year earlier over the study period (*F*_(1, 13)_ = 16.23, *r*^2^_Adj_ = 0.52, *P* = 0.001, Fig. 6b), varying from 26th of March in 2012 for the earliest date over 6 mmHg, to 16th of May for the latest, in 2004. Slope of the latter linear regression implies that the 6 mmHg SD threshold was reached 37.6 days earlier at the end of the study in 2014 than at its very beginning in 2000.

## Discussion

This study is one of the first that documents the monthly abundance and seasonal questing activity of nymphal *Ixodes* ticks by using data from more than a decade (2000–2014). In fact, most follow-up studies of *Ixodes* abundance and questing activity were based on collection of ticks over a few consecutive months, mostly during peak tick activity period (for example [24, 35–38]).

We observed a decrease in *I. ricinus* questing nymph density in the study area, along a south-east-exposed slope of the Chaumont Mountain, North of Neuchâtel (Switzerland), from year 2000 to 2014. The questing nymph density during the spring and autumn peaks displayed the same negative trend. We did not detect any link between decrease in questing nymph density and change in questing activity period. The dates of questing onset O10 (the first day at which nymph density reaches 10% of the peak density, Fig. 1) (26th of February ± 15.2 days), spring peak NP1 (date of spring peak, Fig. 1) (1st of May ± 21.6 days) and autumnal peak NP2 (date of autumnal peak, Fig. 1) (10th of October ± 15.3 days) remained rather stable over years. Hence, the period of questing nymph activity did not significantly change during the fifteen years of nymph follow-up in the study area.

We used climatic data recorded at a station close to the sampling site as well as microclimatic data collected in the field site to examine the role of climatic factors on *I. ricinus* population. Linear regression between the two datasets was used to evaluate the suitability of data recorded at weather station to reflect the actual climatic situation to which the nymphs were exposed [39]. Both climatic databases fitted well and the differences were fairly constant across the data range.

Over the 15 years of study, daily mean values of temperature and RH increased significantly (Climap-net database) (total increase of +0.74 °C and +2.7% for temperature and humidity, respectively). However, no significant change was detected for SD values, probably explained by the fact that RH followed the same positive trend as temperature.

Our results showed that questing nymph density was best explained using climatic data over short time scales (instantaneous field measurements, daily means, 5-days moving averages). Longer time scales (10-, 17- and 30-days moving averages) were slightly poorer predictors, with explanatory power decreasing when time scale increased, suggesting a high reactivity of nymphs to weather conditions. However, as temperature still played a significant role on a 30-days time-scale regardless of the seasonal pattern of activity, it might still be relevant to consider more than one time periods in ecological studies analysing tick questing activity. Our results also pointed out that temperature, and to a lower extent RH, were important predictors even though the model controlled for temporal activity. The most intuitive interpretation is that nymph activity depends on (i) season (date) and (ii) temperature in an additive way. However, the same was not true for SD, which is itself a combination of RH and temperature. Indeed, SD was significantly affecting nymph density only if the model did not account for temporal pattern (date). The interpretation is not straightforward in this case as it seems very unlikely that temporal pattern lead by life-history traits alone can explain most of the variation in nymph activity, regardless of SD. Indeed, the causal link between questing activity and SD was already demonstrated experimentally in controlled laboratory conditions [14] and in semi-natural arenas [13]. One alternative explanation may be that SD actually is the main driver of nymph temporal pattern of activity. Therefore, when time and SD are both included in the same model, they tend to explain the same part of the variation. This explanation is supported by the high correlation observed between SD and time (date). However, to properly disentangle the relative contribution of SD, temperature, RH and seasonality *per se*, more experimental work would be useful. Finally, NBGLMs results found no effect of precipitations on nymph density, as also suggested by Estrada-Peña et al. [39], but which contrasts with the results of Hayes et al. [40].

Additional analyses showed the probable existence of lower and upper thresholds below and above which the questing activity of nymphs was limited, which was already indicated by the significance of the quadratic term of temperature in the NBGLMs. This seemed to be especially true for temperature and SD, although the precise role of both variables in nymph activity and phenology might be slightly different and potentially confounding, as discussed above. For example, the number of hot days (5-day moving average of maximal temperature > 27 °C) showed a trend for a negative correlation with CND2, but the number of such days did not change significantly over the study period and could not therefore explain the observed decrease in tick density.

Most notably, considering SD, we observed that questing nymph density increase in spring was strongly limited by an upper threshold of a SD value of 6 mmHg on 2-days moving average. Moreover, during the study period, this upper limit of SD value was exceeded each year earlier over the 15 study years, limiting the period duration during which nymph may benefit from favourable weather conditions for questing. Indeed, we observed that this limit was reached around 37 days earlier in 2014 than it was at the very beginning of the study in 2000. As SD slightly increases in spring and shows highest values in summer, the period of time from 1st of January to the first day when the 6mmHg threshold is reached informs us about how nymphs may be restricted in their questing behaviour, and how this change may eventually limit their survival [14–20]. A possible mechanistic explanation of the link between SD and questing behaviour is related to the tick resources. Indeed, *I. ricinus* ticks use their fat supplies to quest for hosts on vegetation and to maintain their water balance. Van Es et al. [41] and Randolph & Storey [13] have shown that the rate of lipid consumption increases under unfavourable conditions of temperature and humidity, respectively. It was reported in UK and Switzerland that nymphs collected in spring possessed less fat content (energy reserves) than their autumn counterparts [42, 43] which illustrates the vulnerability of nymphs that have to face unfavourable conditions of SD values in spring.

As we also showed that early exceeding of these values correlated with lower nymph density over the year, we expect that the observed trend affects negatively tick population by reducing the duration of favourable period for questing nymph activity and development.

Surprisingly, we did not detect any modification in questing nymph activity period - chiefly the dates of nymph questing onset, and of spring/autumnal peak density during the study period. Most probably other factors influence seasonality of nymph activity, as for example winter temperature. In fact, the average temperature during January and February was correlated with the number of days from 1st January to onset date of tick presence O10, cold temperatures delaying questing nymph activity onset. But this factor did not induce any change in O10 over years.

Alternatively, it is possible that the period of nymph activity actually changed, but the available data may not have been sufficient enough to detect it, as those analyses concerned only 15 data points, corresponding to the 15 study years. This possibility is supported by the great variation in questing onset and peak dates that may have contributed to hide a more global trend. Moreover, more complex changes in nymph activity might exist without impacting global activity period. For example, activity period may be identical today but with fewer nymphs, e.g. only those being in suitable microhabitats, being able to quest during particularly dry or hot days. In that case, after SD threshold is exceeded, the only observation is a slow-down in nymph increase without any necessary change in nymph onset or peak date.

These results also suggest that further studies should pay more attention to other factors influencing tick life-cycle and phenology over long time, including the importance of suitable microhabitats in tick population resilience, but also host density. These factors as well as climatic variations should be assessed over more than one year, as bad conditions might affect tick population over several years, due to the initial reduction of population.

The absence of change in nymph activity period is also surprising regarding to a commonly feared scenario about global warming suggesting that warmer winters may allow ticks to quest earlier for new hosts (e.g. [23, 44]). However, we did not find any trend supporting the hypothesis of anticipated questing behaviour over years, or significant change in late winter temperatures. Although we found an interpolated increase of temperature of 0.74 °C over 15 years at the MeteoSwiss station of Neuchâtel, our present results suggest that because of the probable importance of temperature and SD thresholds, stochastic weather variation may be more important than global trends as tick density driver (see also [37]). Note that our results are limited to this location and time interval and should be considered carefully before linking them to global warming.

Climate change may therefore play a stronger role by increasing extreme events during which lower or upper thresholds, like temperature or SD, are rapidly exceeded, disturbing nymph activity. This sensitivity to very hot/cold or dry periods may be deleterious to tick population at low altitudes, overriding the positive effects that warmer winters may eventually have in the long run. For example, a survival study in the laboratory reported that frequent temperature changes in winter threaten tick survival more importantly than very low temperatures [45].

Despite our efforts to collect ticks each month over 15 years (more than 50,000 collected nymphs) at one site by one person, we consider that large part of nymph density and phenology variation remains difficult to explain. As discussed above, at least two additional key points should be addressed to better understand tick populations: (i) assess to which extent unfavourable conditions determine tick population also for one or two years after the extreme climatic event, and (ii) to evaluate the presence and activity of tick hosts in the sampling area, and their susceptibility to climate variations, possibly amplifying the effects of extreme events on tick population. To have a better and more complete overview of the changes of nymph density and phenology, these critical points should be investigated in further studies. Most importantly future research endeavours should consider collecting data on tick abundance and seasonal questing activity over long-term and without interruption (during the whole year). The current situation with very limited number of such studies does not help to properly disentangle the main factors that influence tick population.

## Conclusions

Data collected over 15 years of monthly field samplings revealed a decrease in questing *I. ricinus* nymph abundance in a Swiss deciduous forest from 2000 to 2014. Our results indicate that a gradual modification in the annual pattern of air SD may have contributed to the observed decline. More globally, our results suggest that nymphs are reactive to rapid changes in their environment, and may be particularly sensitive to extreme climatic events, which should be considered as an important driver of tick population dynamics. Further studies assessing the impact of host abundance and the role of microhabitats in *I. ricinus* phenology and questing behaviour would strengthen our understanding of the ecological factors affecting tick density. The present work can be considered as a coherent case study addressing the question of tick density variation in a changing climate context.

## Additional files

Additional file 1: **Figure S1.** The sampling area with the circular footpath. **Figure S2.** The sampling area with the maximal temperature threshold for nymph activity. **Figure S3.** The sampling area with the cumulated nymph density for spring semesters. **Figure S4.** The sampling area with the cumulated nymph density for autumn semesters. **Figure S5.** The sampling area with the linear correlations between Climap-net data and field measures for temperature and humidity. **Figure S6.** Parallel plots of nymph density and relevant climatic variables (1 plot by year). **Figure S7.** Parallel plots of nymph density and the number of days of each year during which the upper threshold of maximal temperature was reached. (DOCX 8766 kb)

Additional file 2: **Table S1.** Descriptive statistics of nymph density for spring semesters. **Table S2.** Descriptive statistics of nymph density for autumn semesters. (DOCX 19 kb)

## Abbreviations

CND1: Cumulated nymph density for the first semester
CND: Cumulated nymph density for the whole year
CND2: Cumulated nymph density for the second semester
GLM: Generalized linear model
GLS: Generalized least squares
LR: Likelihood ratio test
NBGLM: Negative binomial generalized linear model
NP1: Maximum nymph density for spring peak
NP2: Maximum nymph density for autumnal peak
O10: Nymph activity onset date
PND1: Spring peak date
PND2: Autumnal peak date
PR: Precipitations
RH: Relative humidity
SD: Saturation deficit
TBE: Tick-borne encephalitis
