# Supplementary material for: Influence of climatic factors on Ixodes ricinus nymph abundance and phenology over a long-term monthly observation in Switzerland (2000–2014)
Source: Parasit Vectors. 2018 May 8;11:289. doi: 10.1186/s13071-018-2876-7 (PMC5941567; doi:10.1186/s13071-018-2876-7)
Supplement: Supplementary file 1 — Figure S1. The sampling area with the circular footpath. Figure S2. The sampling area with the maximal temperature threshold for nymph activity. Figure S3. The sampling area with the cumulated nymph density for spring semesters. Figure S4. The sampling area with the cumulated nymph density for autumn semesters. Figure S5. The sampling area with the linear correlations between Climap-net data and field measures for temperature and humidity. Figure S6. Parallel plots of nymph density and relevant climatic variables (1 plot by year). Figure S7. Parallel plots of nymph density and the number of days of each year during which the upper threshold of maximal temperature was reached. (DOCX 8766 kb) [file 13071_2018_2876_MOESM1_ESM.docx]

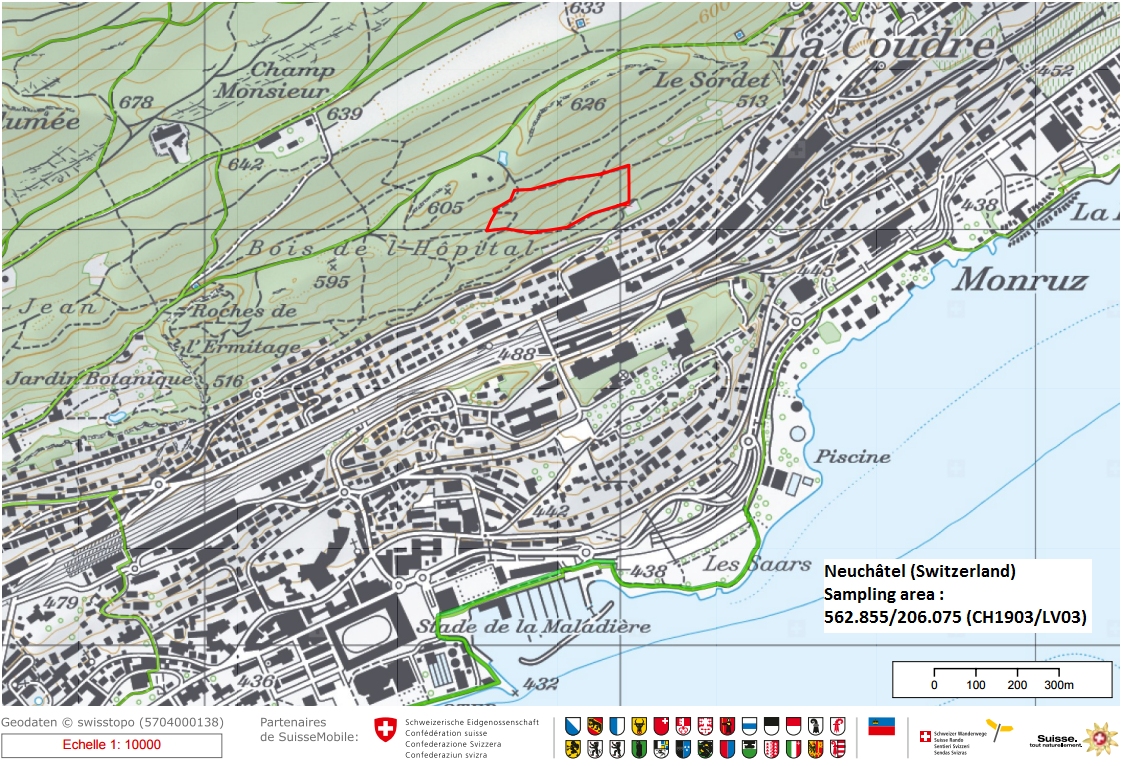


**Figure S1**. Map (Swisstopo) 1 :10'000 of the sampling area (in red) in Neuchâtel (Switzerland), Bois de l’Hôpital. CH1903: Swiss grid coordinates: 563017/206063; 562650/205987; 562764/206105; 562975/206140


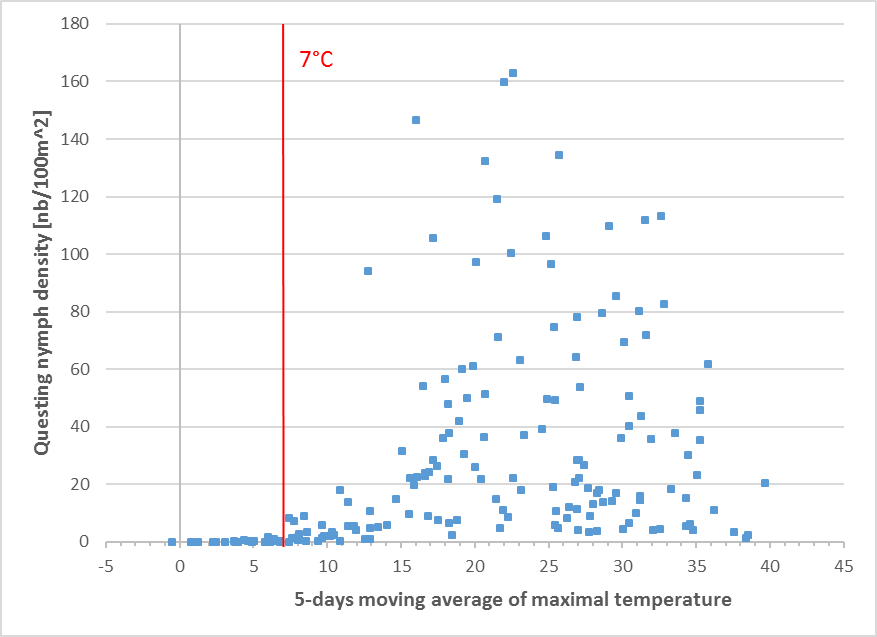


**Figure S2**. Correlation between the 5-day moving average of maximal temperature and questing nymph density, showing the limit of 7°C, below which questing nymphs were rarely collected in the field. N=173.


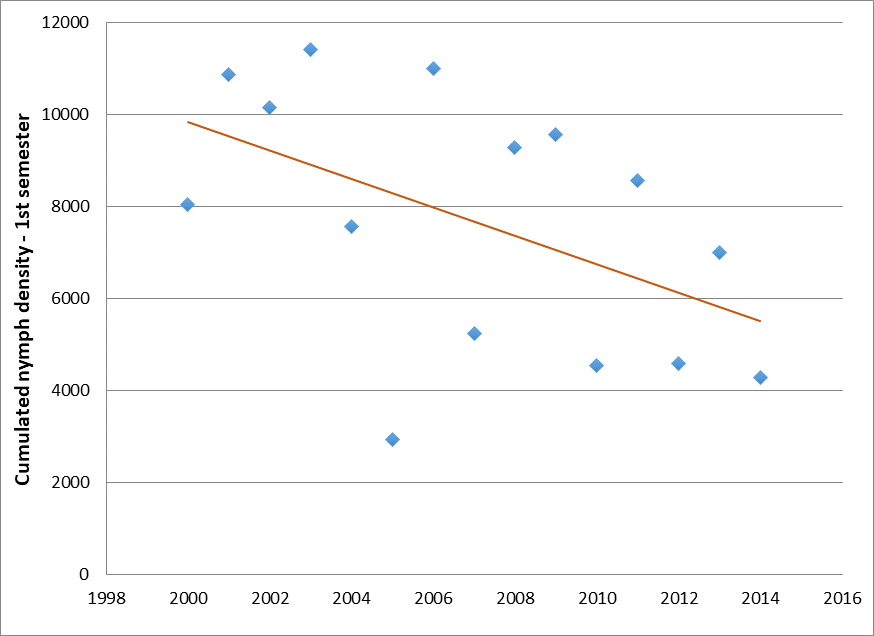


**Figure S3**. Cumulated questing nymph density during the 1st semester (CND1, January 1^st^ to June 30^th^) from 2000 to 2014.


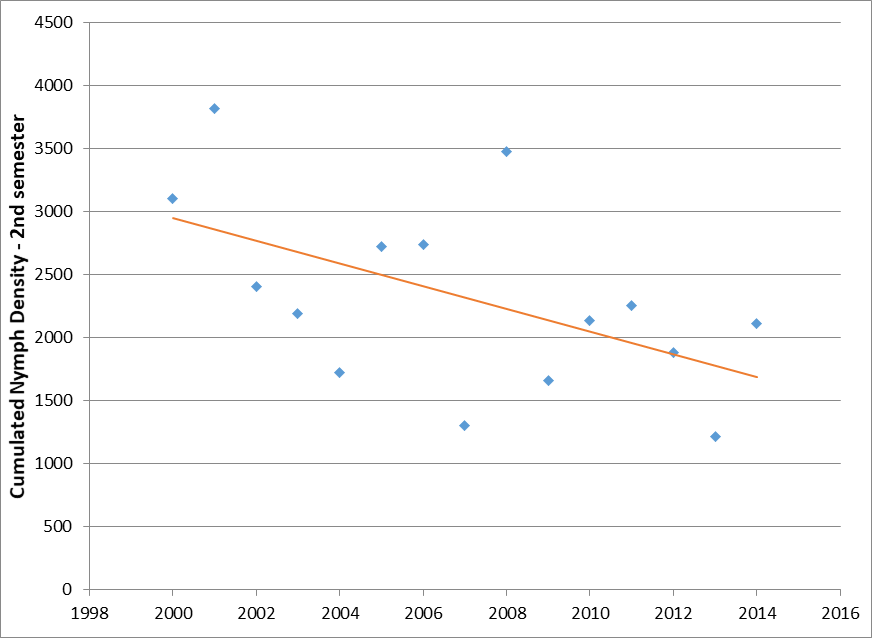


**Figure S4**. Cumulated nymph density during the 2nd semester (CND2, July 1^st^ to December 31^th^) from 2000 to 2014


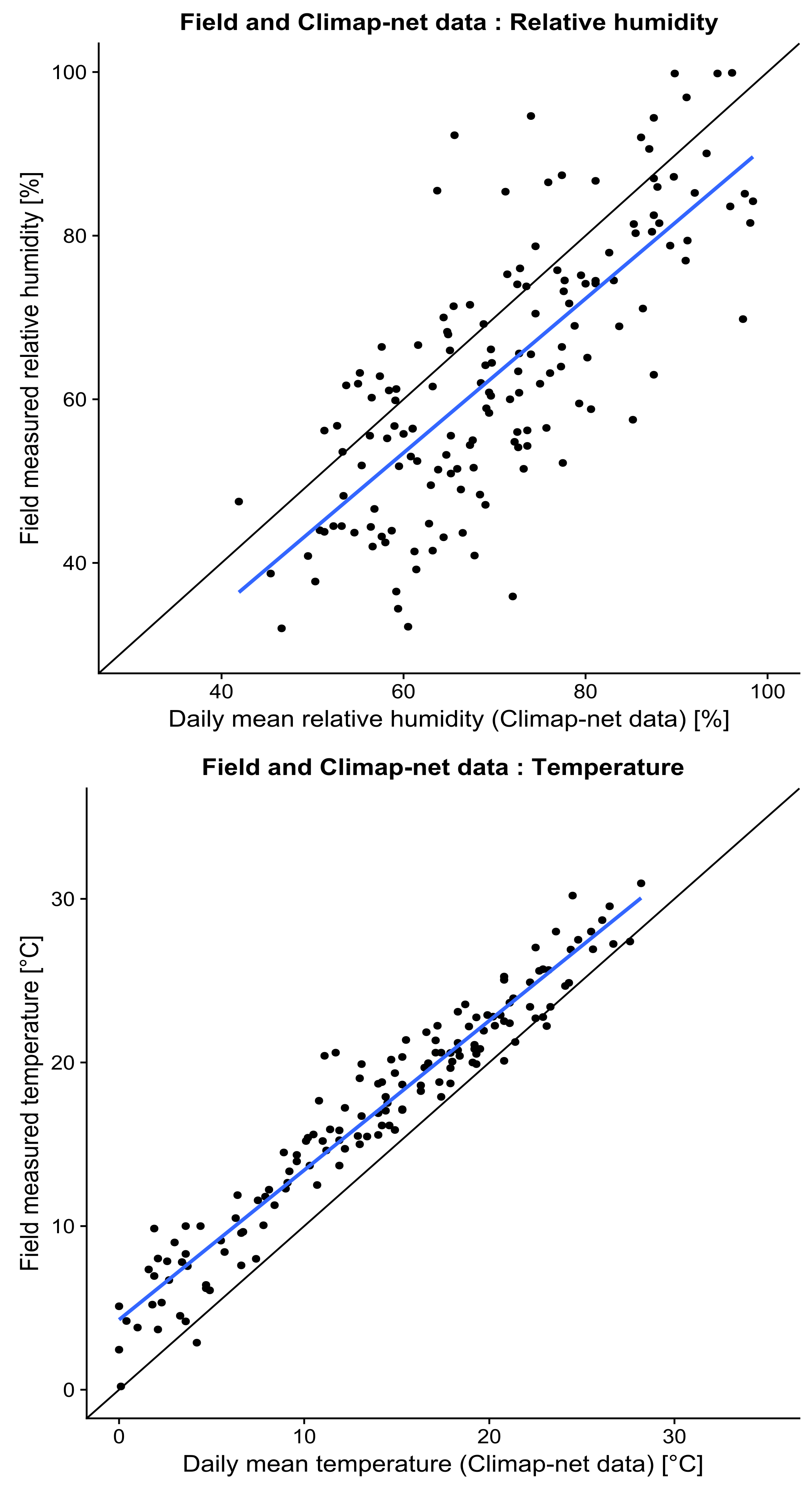


**Figure S5**. Linear correlation between climatic measurements in the field (y axes) at sampling site (altitude: 520m) and daily mean values of the same day obtained from the Swiss Meteorological Centre station (x axes) in Neuchâtel (altitude: 485m). Upper plot shows relative humidity and lower plot shows temperature. Each point represents a sampling date. Blue line is the linear regression between data points, black line is the 1:1 slope.


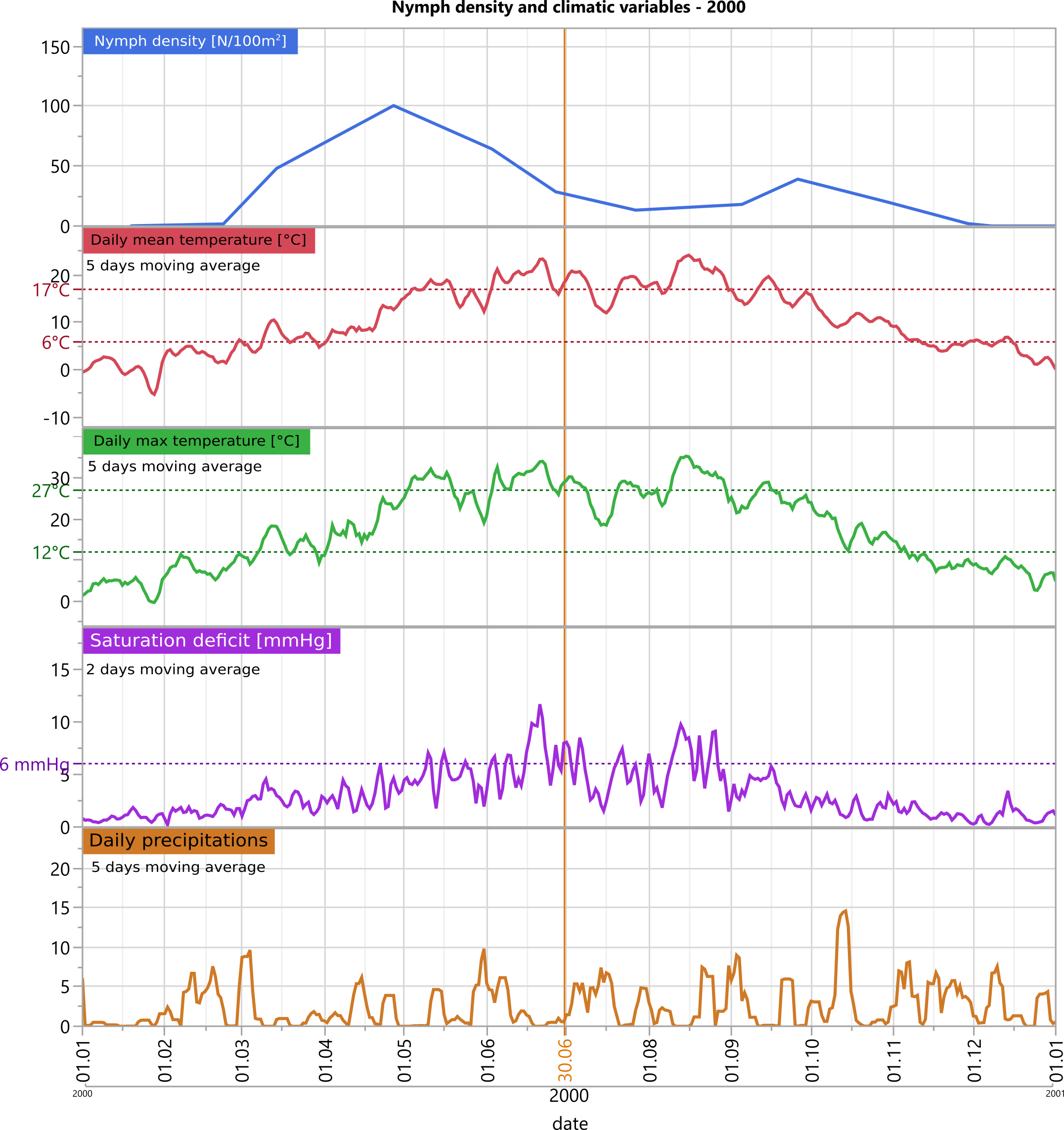


**Figures S6**. Parallel plots of nymph density and climatic variables from year 2000 to 2014. Blue line: questing nymph density (data points include fictive null samples (see Tick Density Parameters, in Material and Methods), red line: 5 days moving average of the daily mean temperature, green line: 5 days moving average of the daily maximal temperature, purple line: 2 days moving average of the daily mean of saturation deficit and orange line: 5 days moving average of the daily sum of precipitations in millimeter.

Horizontal dotted lines show, when relevant, possible thresholds linked to questing nymph activity that were visually assessed and arbitrary chosen to improve reading. Unlike temperature and saturation deficit, no clear impact of precipitations on tick phenology was discerned.


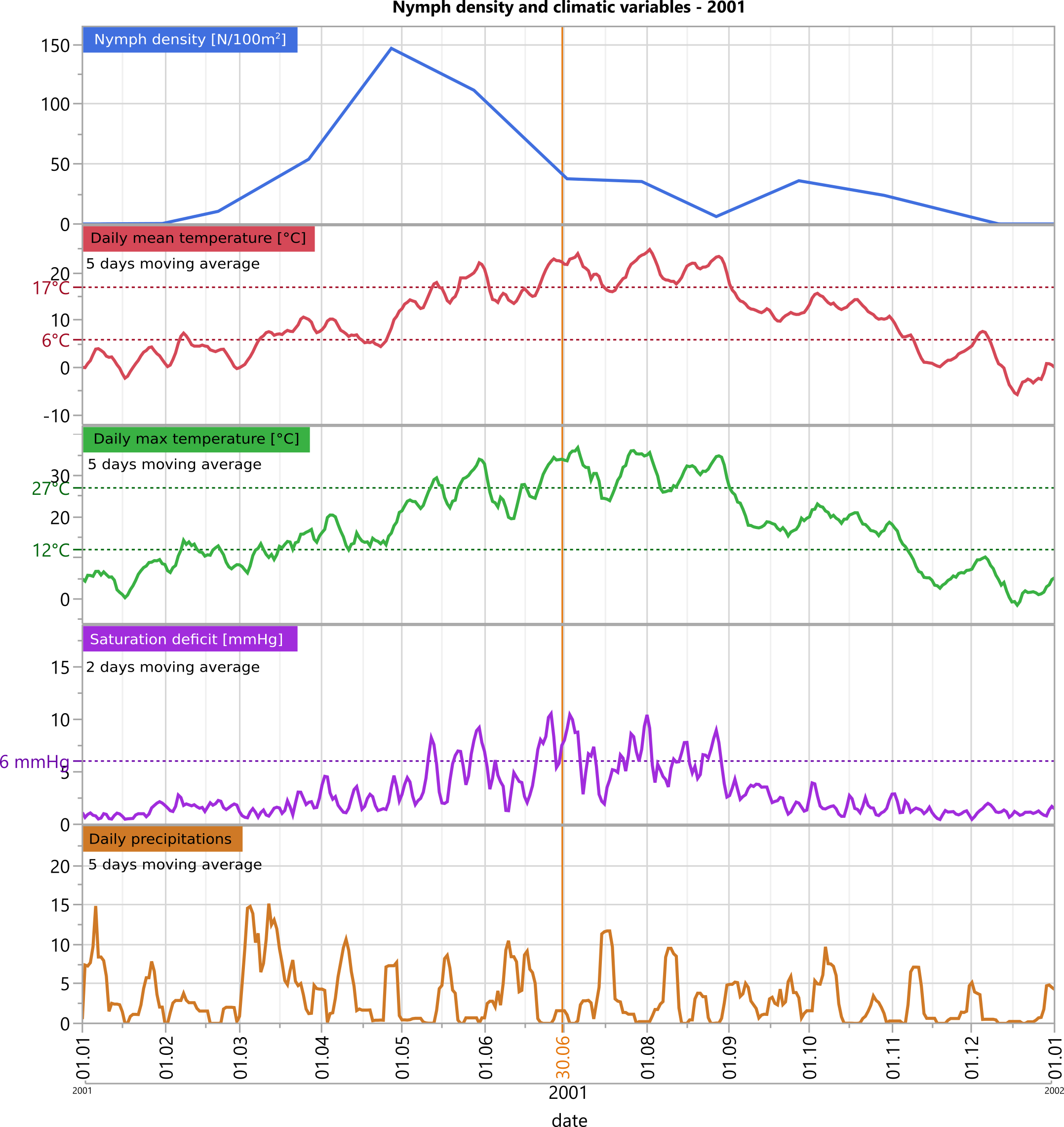


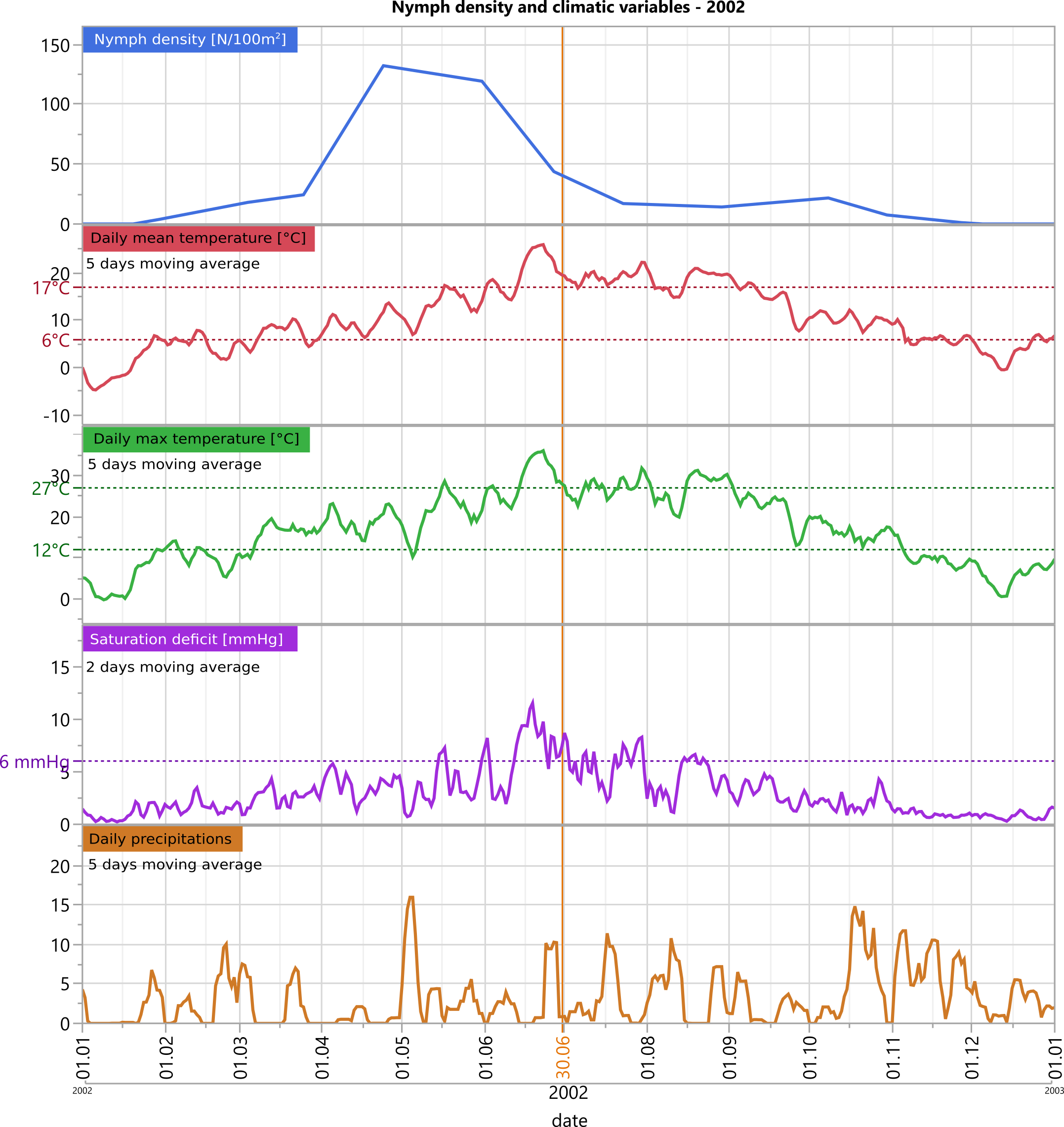

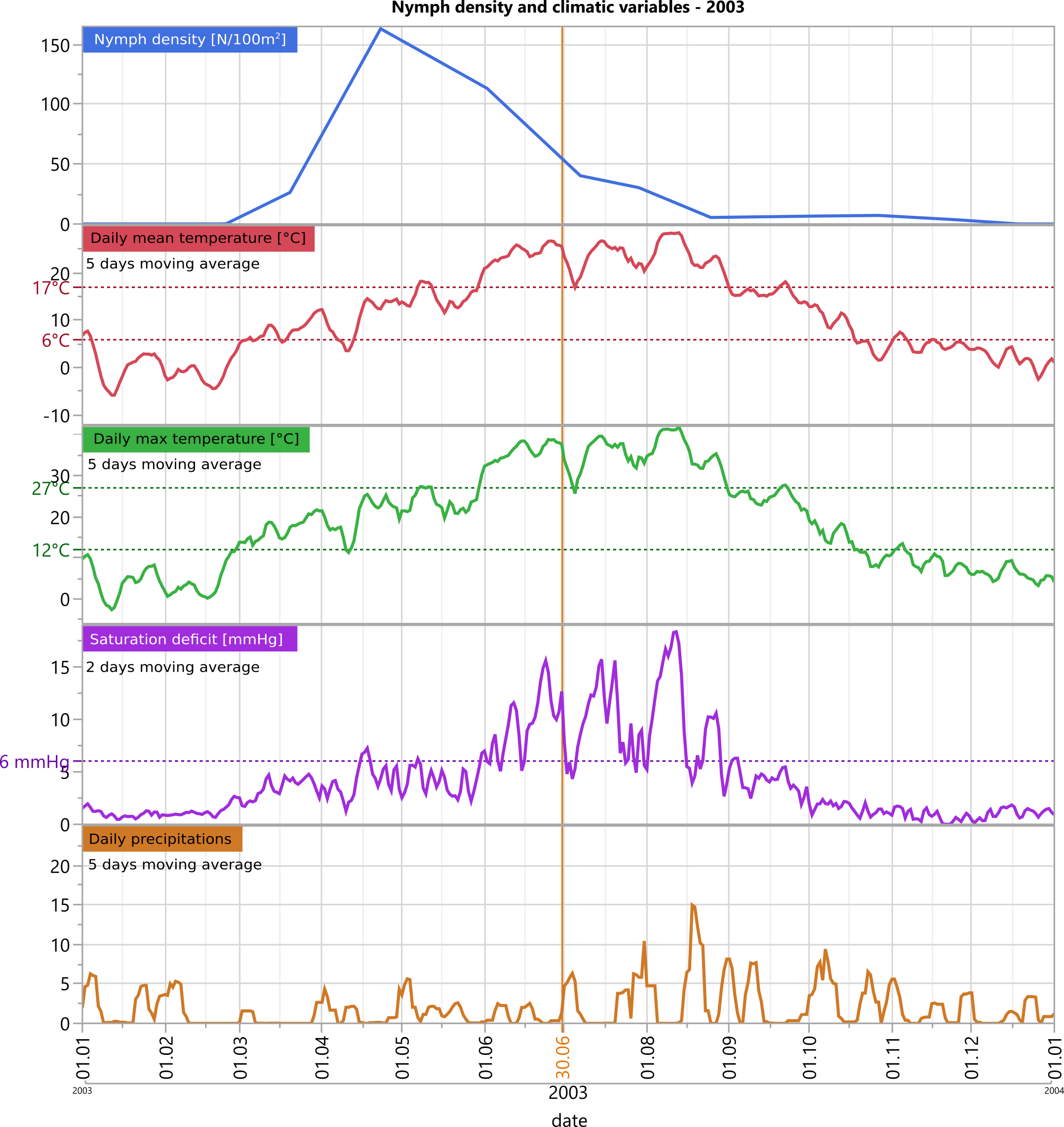


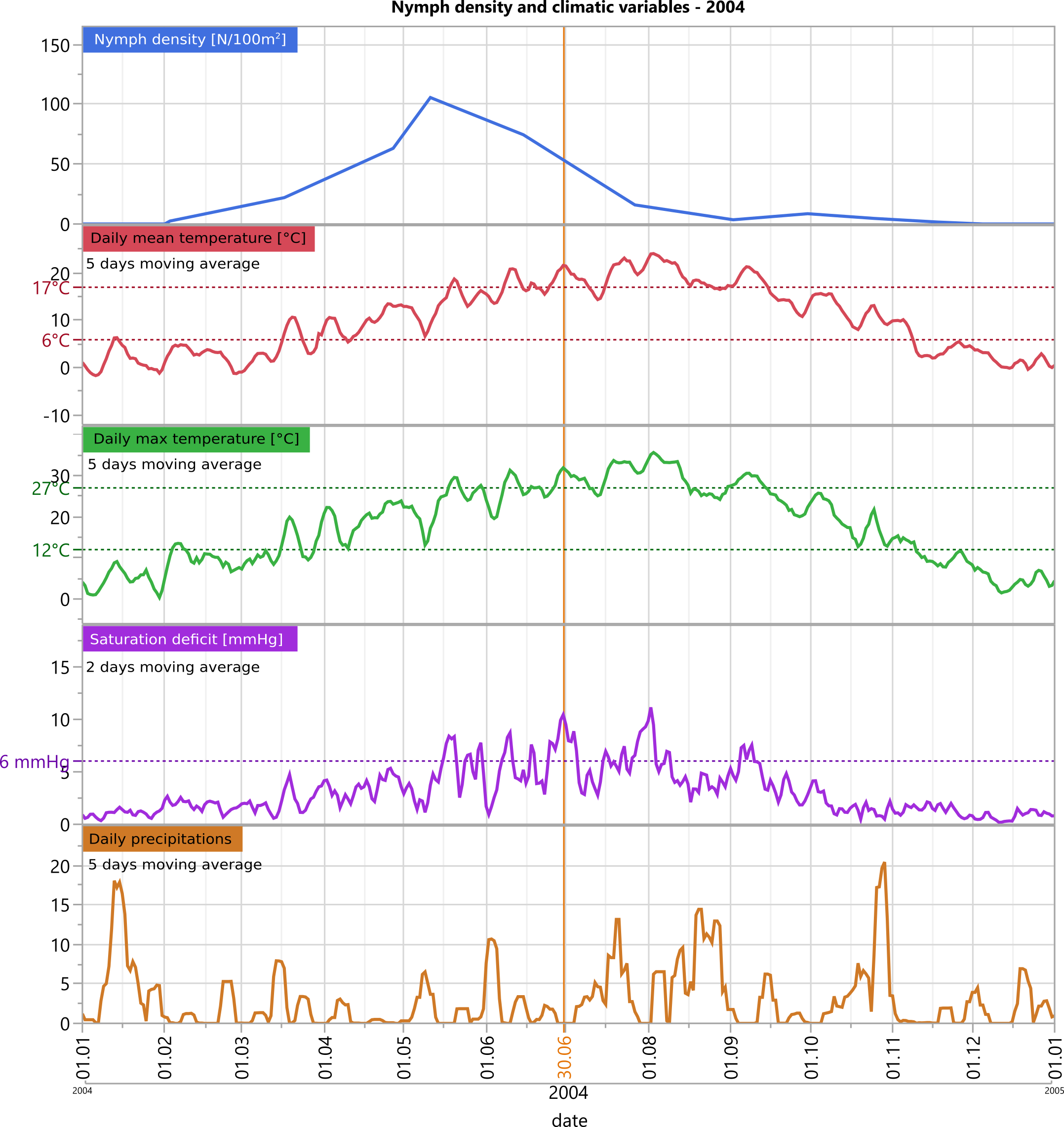


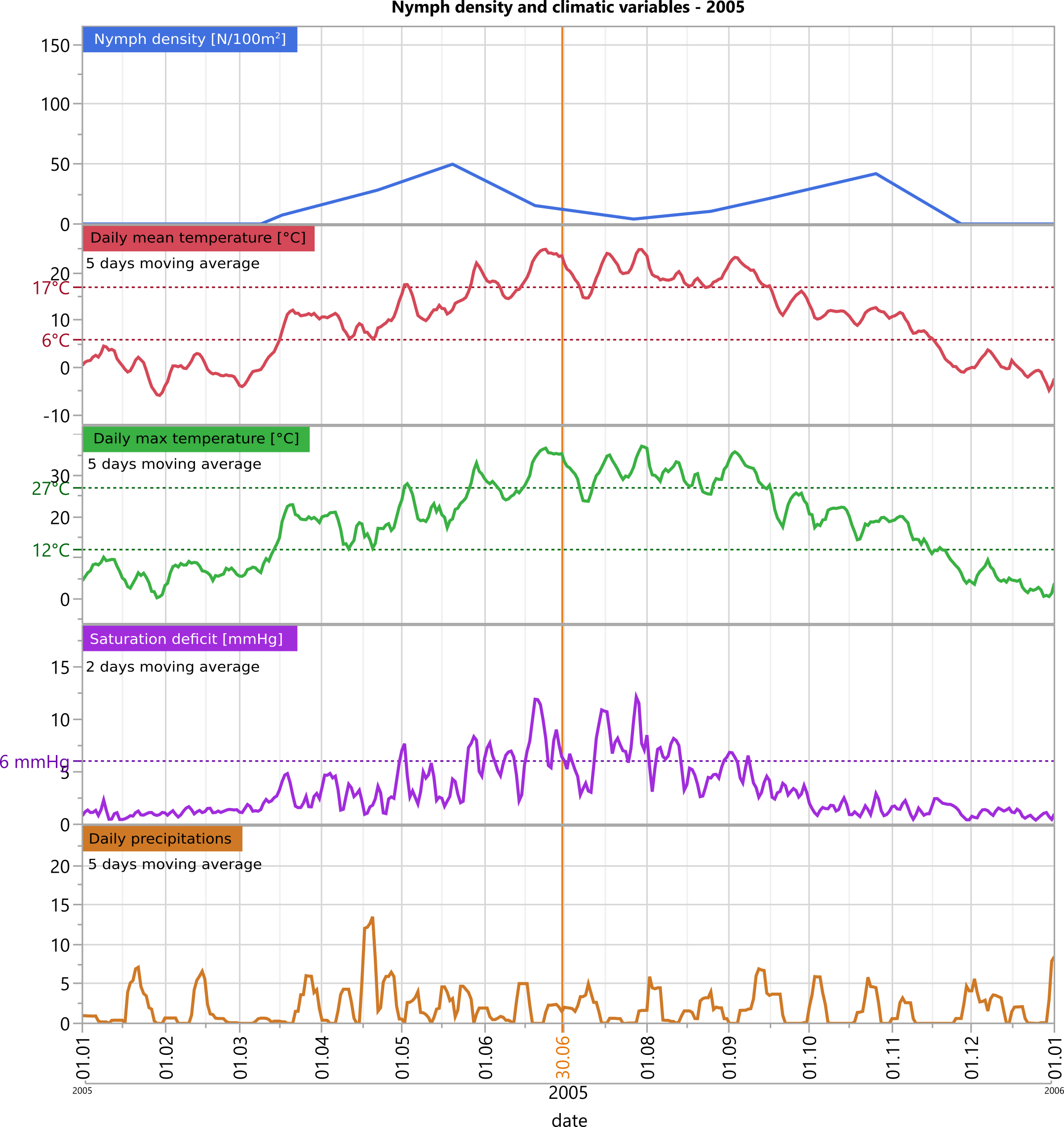

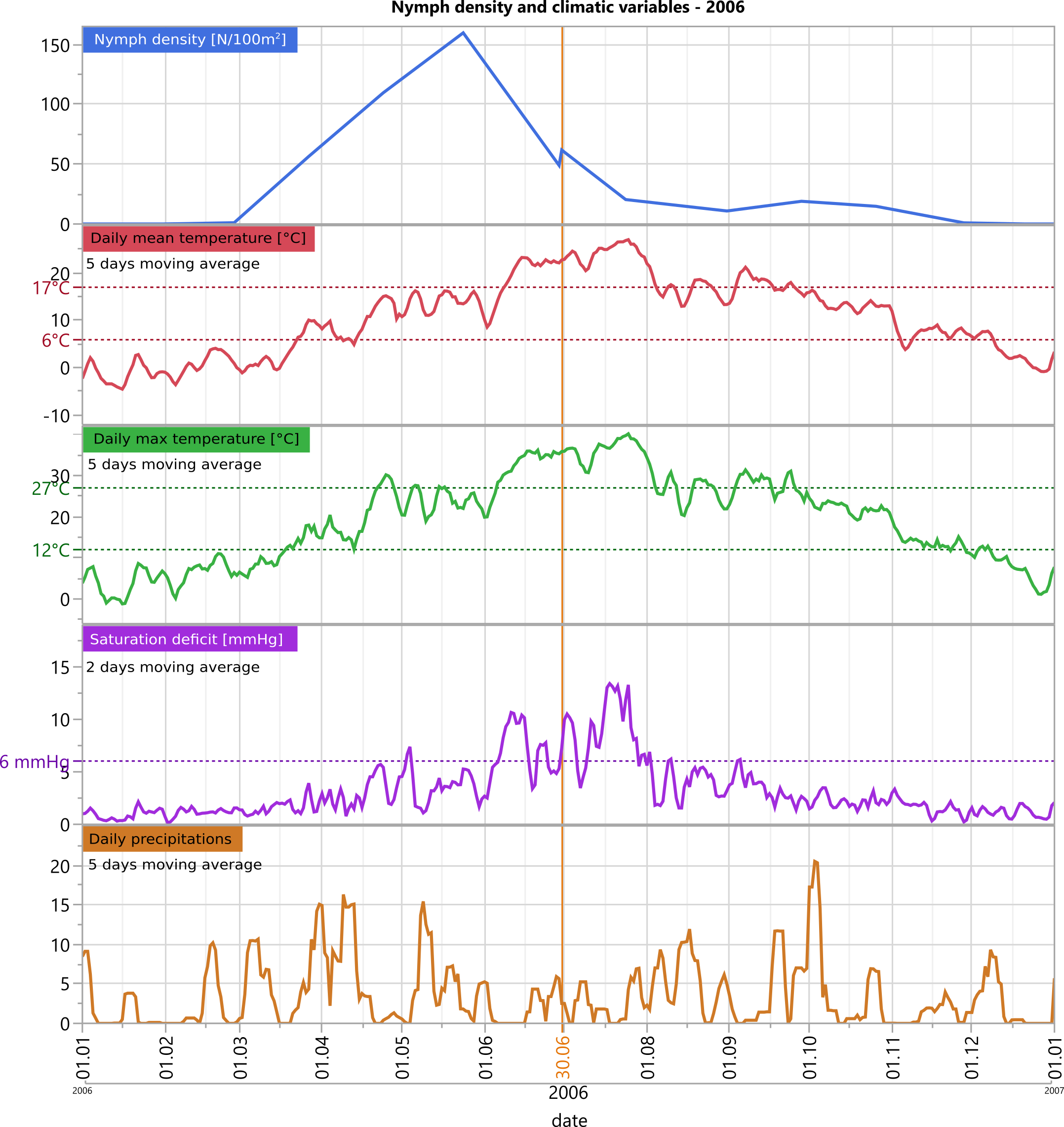


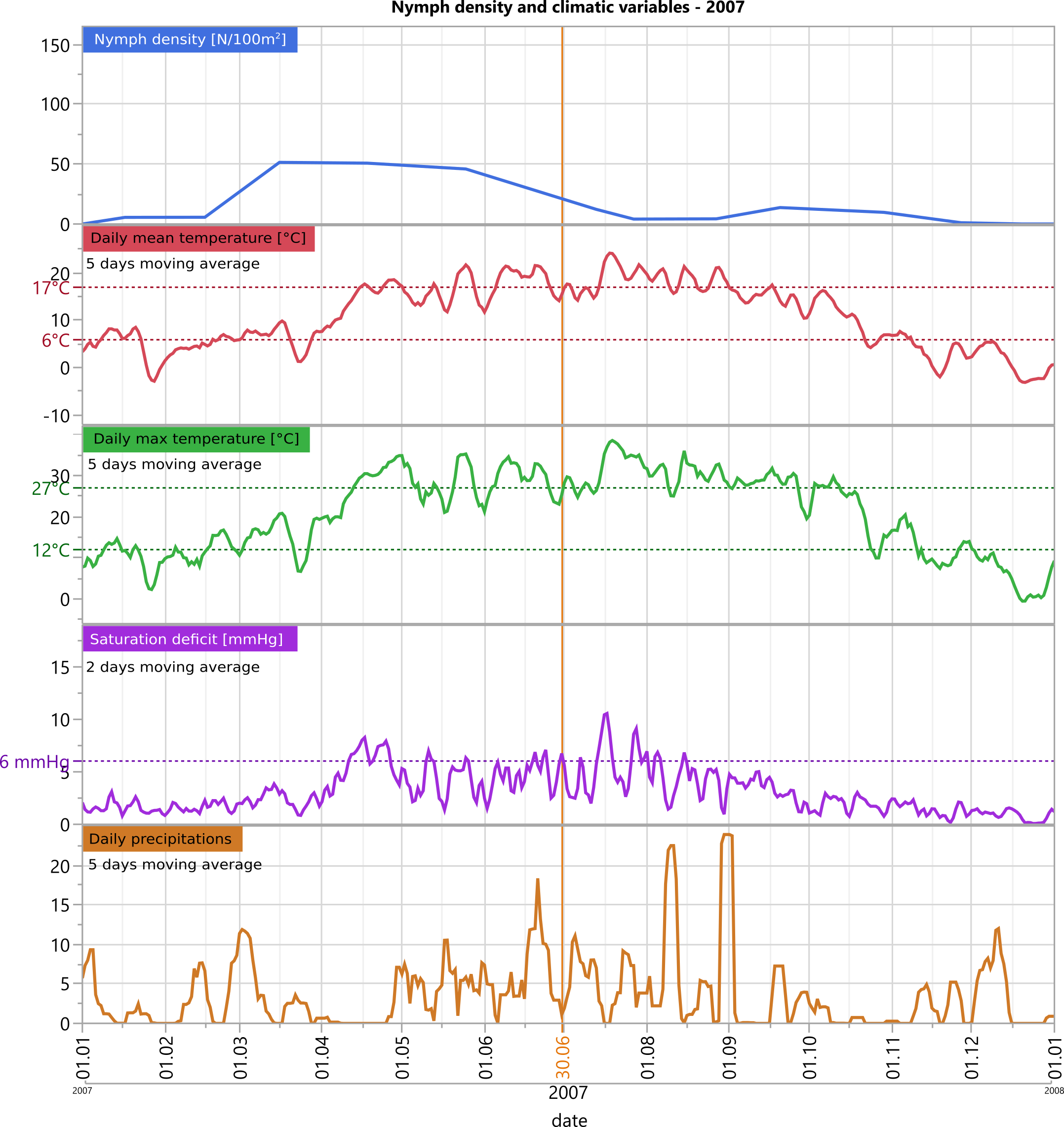


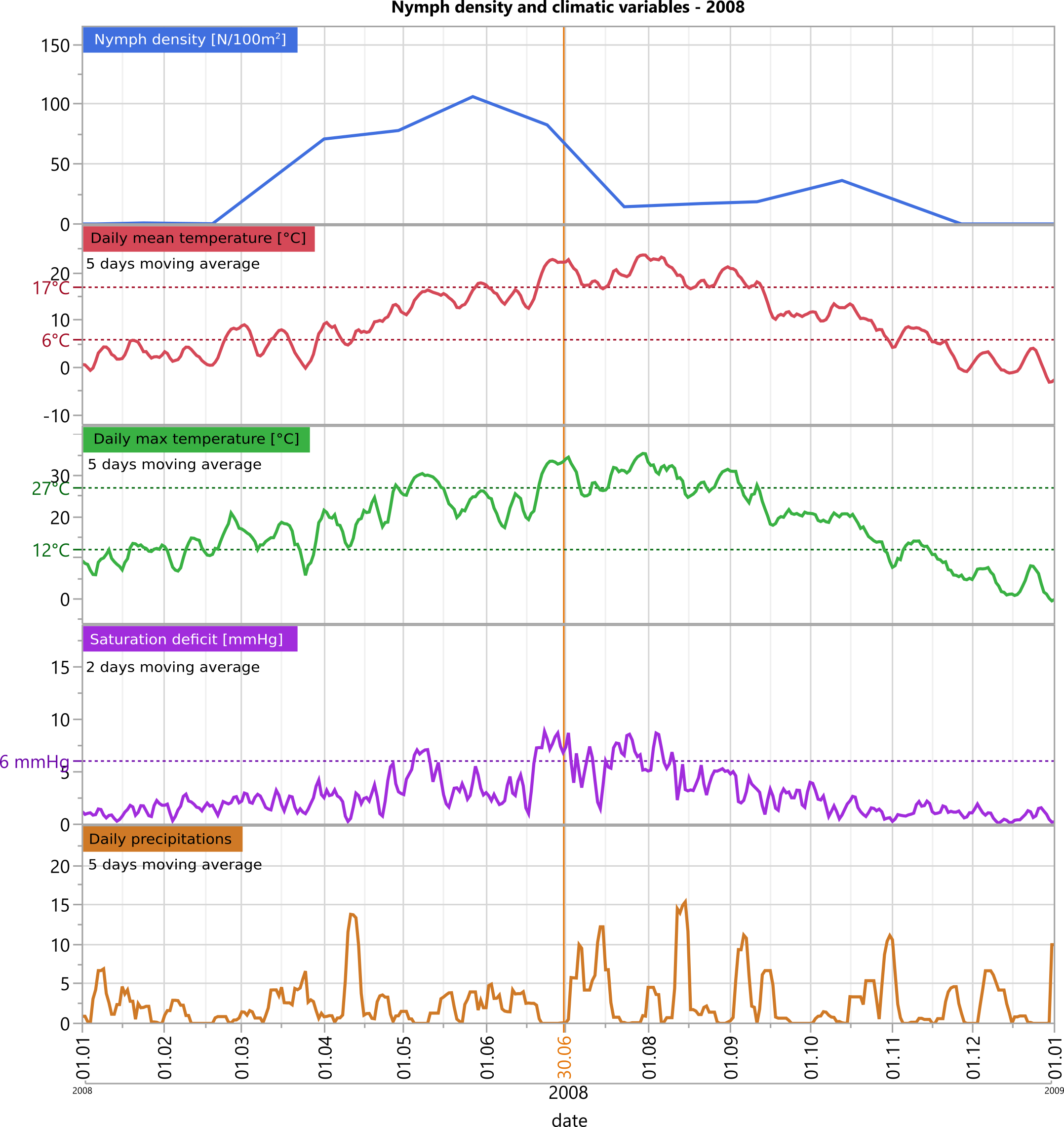


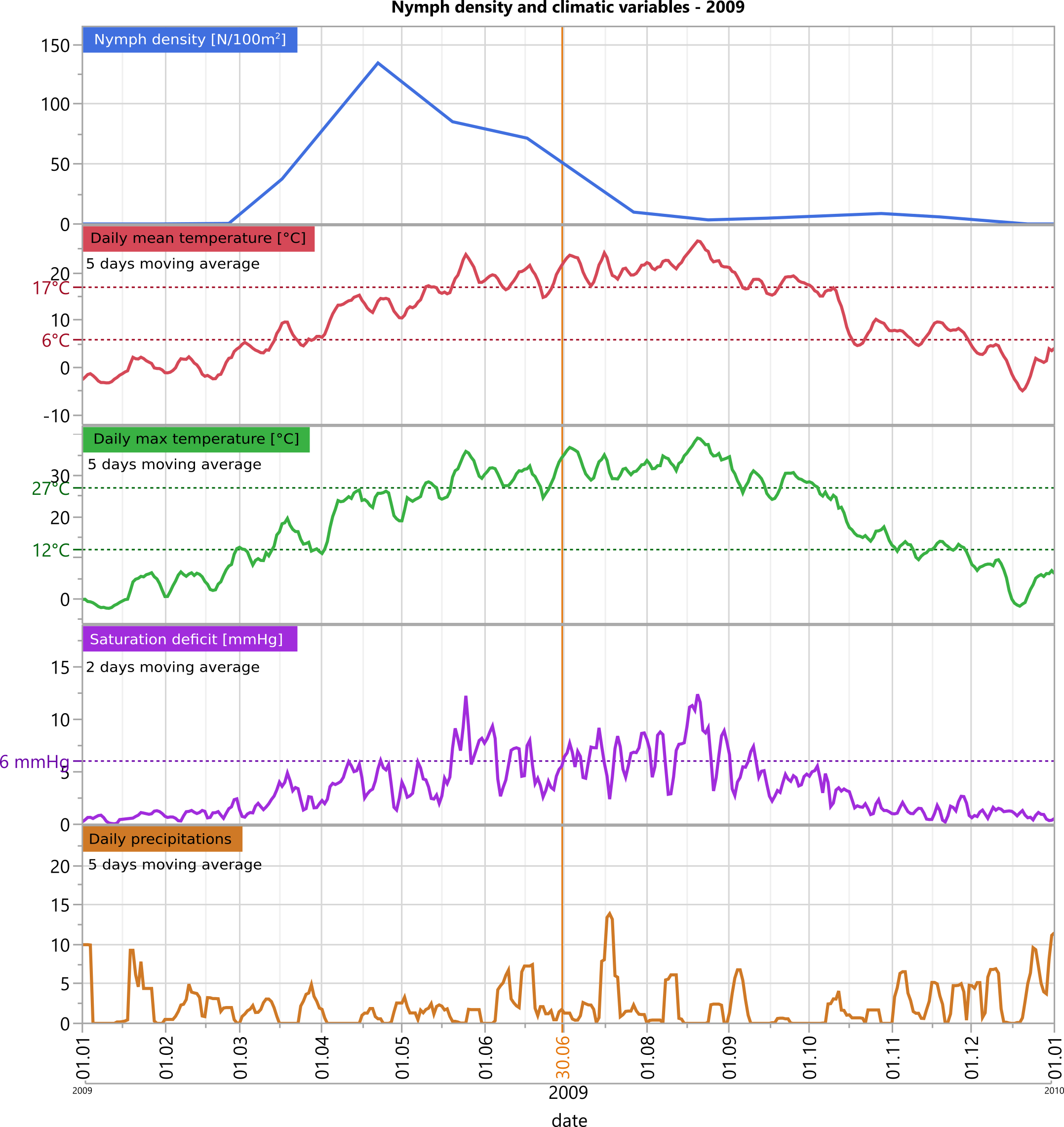


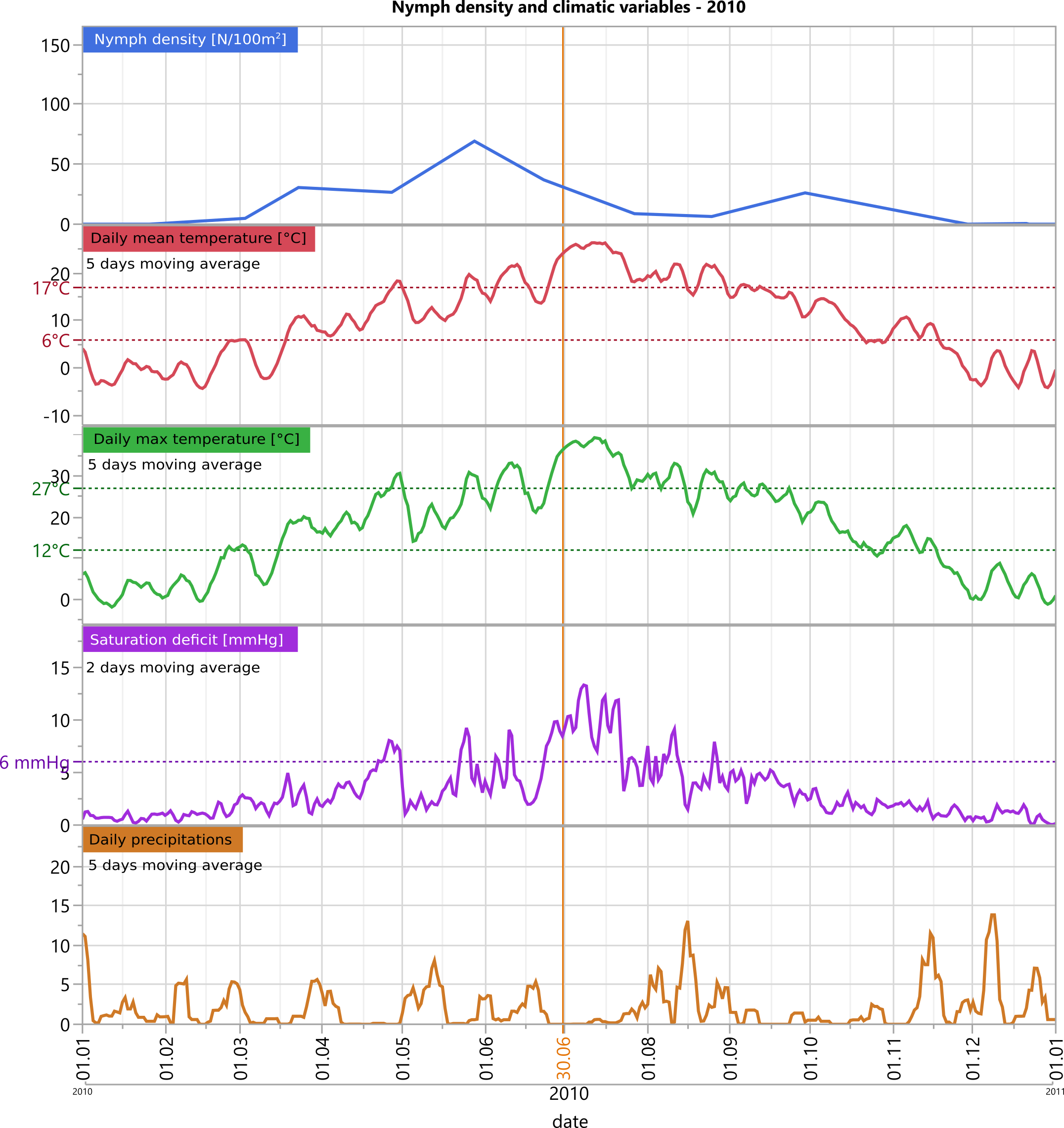


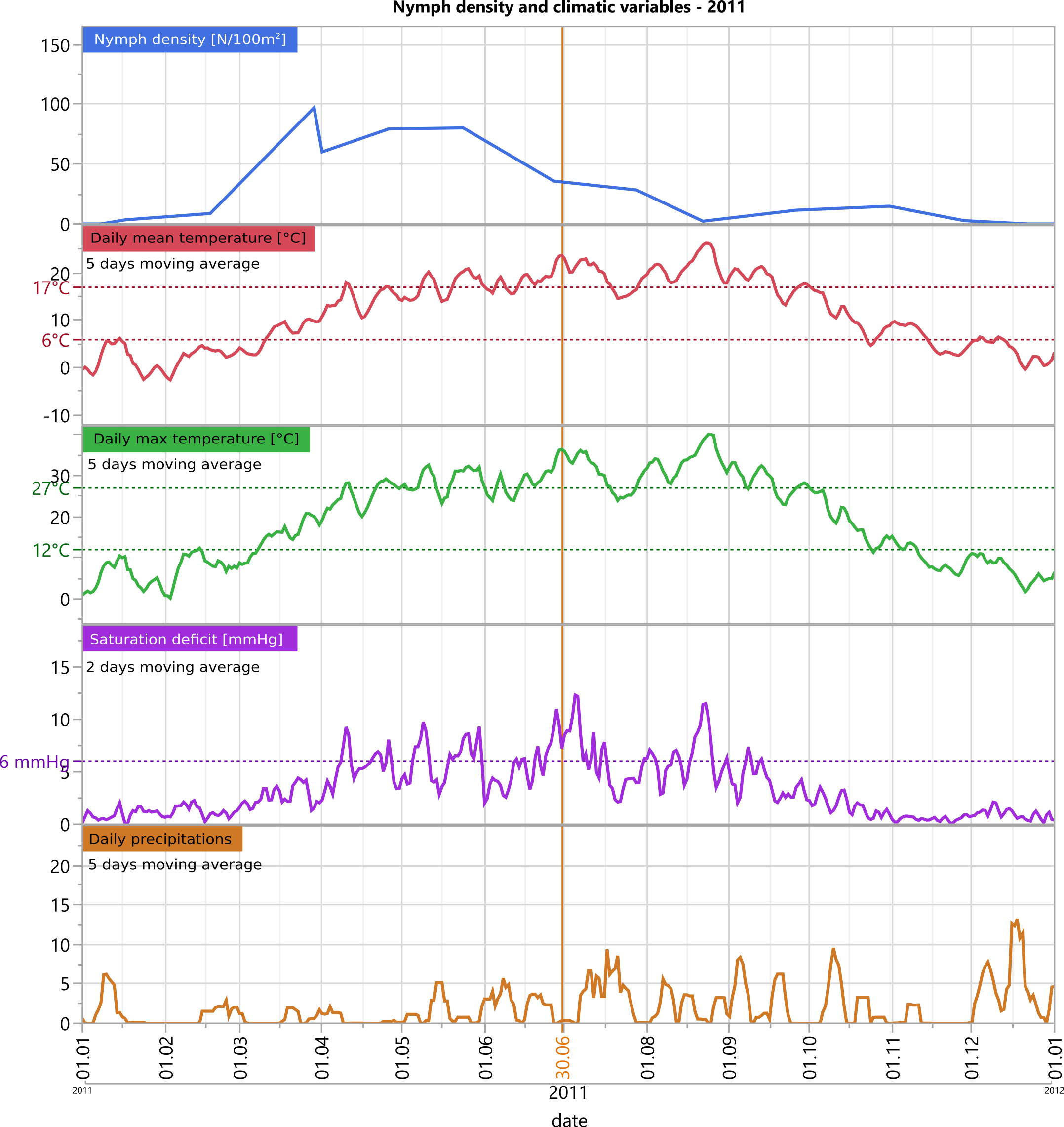


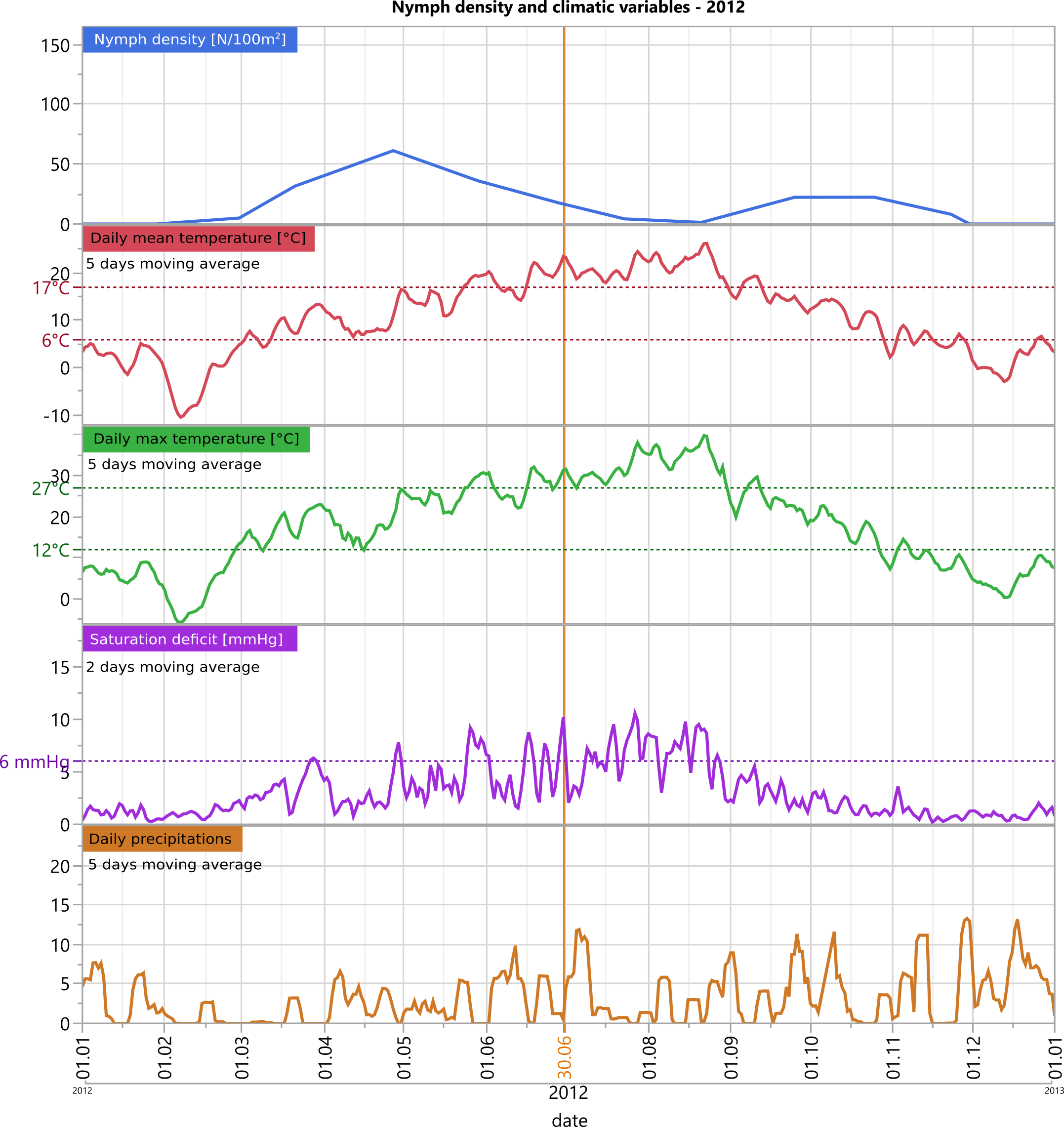


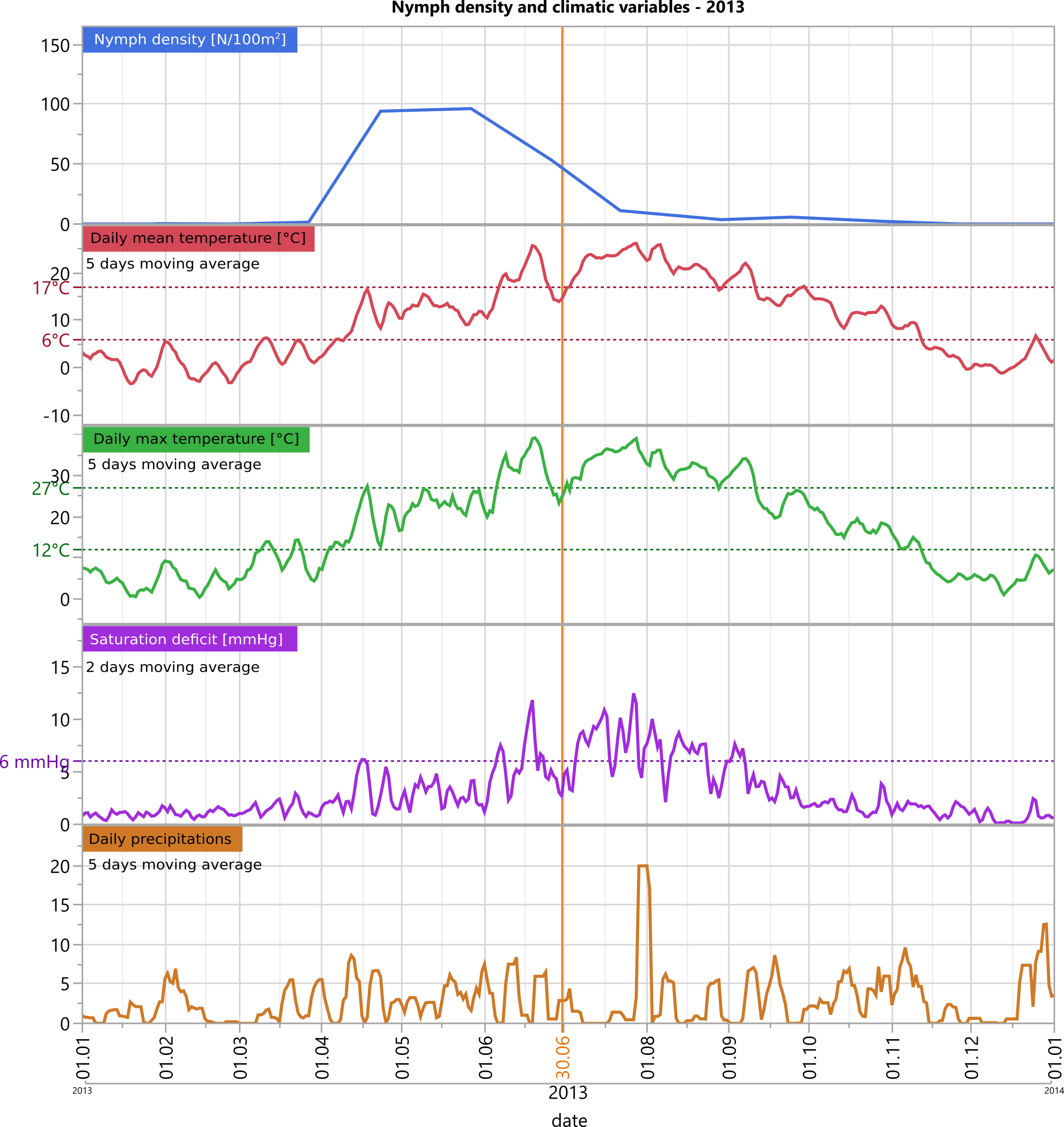


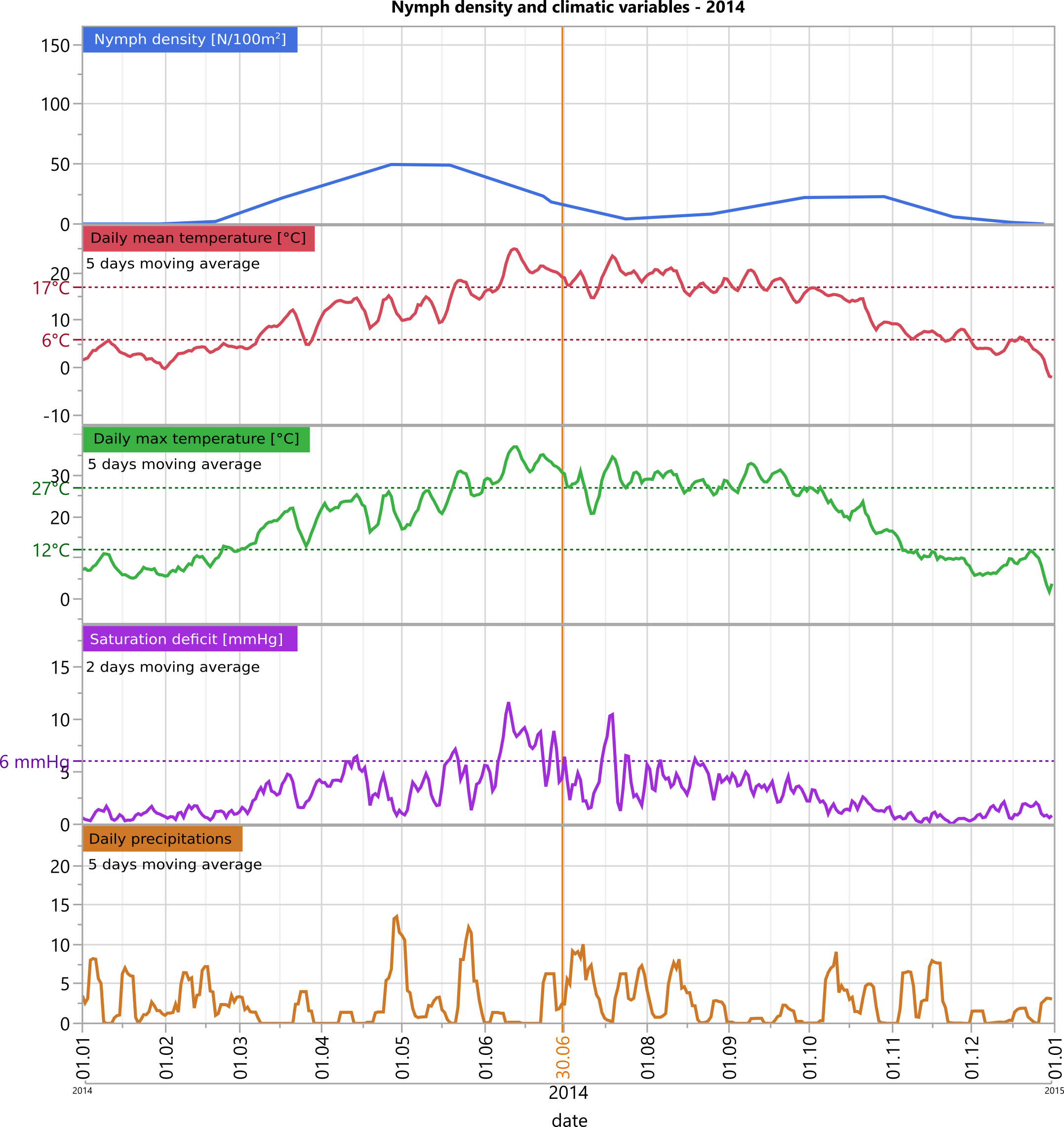


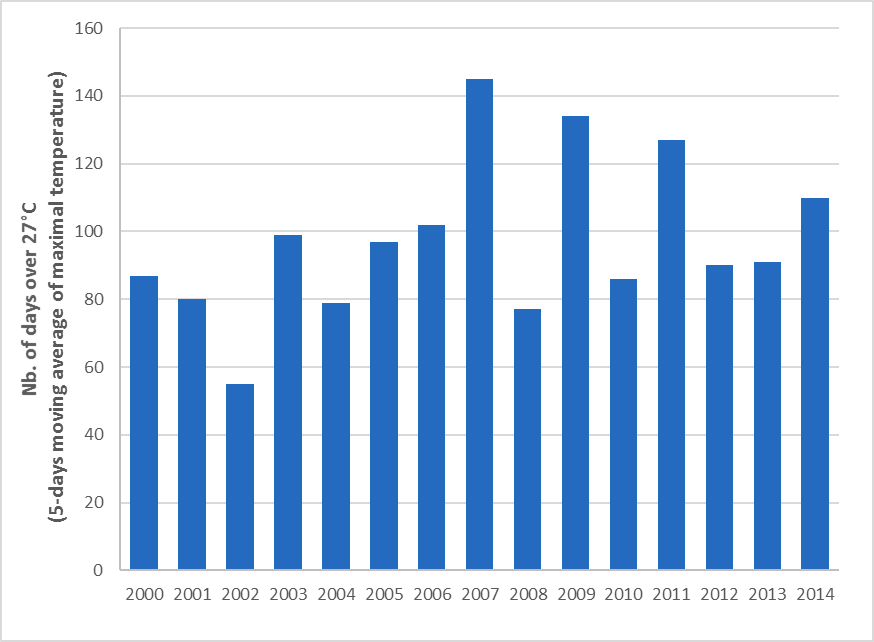


**Figure S7**. Annual variation of the number of days displaying a 5-day moving average of maximal temperature higher than 27°C.
