# Supplementary material for: Influence of climatic factors on Ixodes ricinus nymph abundance and phenology over a long-term monthly observation in Switzerland (2000–2014)
Source: Parasit Vectors. 2018 May 8;11:289. doi: 10.1186/s13071-018-2876-7 (PMC5941567; doi:10.1186/s13071-018-2876-7)
Supplement: Supplementary file 2 — Table S1. Descriptive statistics of nymph density for spring semesters. Table S2. Descriptive statistics of nymph density for autumn semesters. (DOCX 19 kb) [file 13071_2018_2876_MOESM2_ESM.docx]

**Table S1**. Mean, maximal, minimal and cumulated (CND1) questing nymph densities of the first semester of each year, given in number of nymphs per 100m^2^. For this latter measure only (CND1), fictive null samplings were considered (see Tick sampling part in Material and Methods for details).

| **Year** | **Mean density** | **Maximal density** | **Minimal density** | **CND1** |
| --- | --- | --- | --- | --- |
| 2000 | 40.53 | 100.48 | 0.19 | 8041.373 |
| 2001 | 53.94 | 146.71 | 0.10 | 10875.99 |
| 2002 | 56.93 | 132.22 | 3.91 | 10155.77 |
| 2003 | 75.74 | 163.11 | 0.19 | 11422.5 |
| 2004 | 53.57 | 105.62 | 2.48 | 7572.276 |
| 2005 | 20.23 | 49.95 | 7.44 | 2922.406 |
| 2006 | 62.50 | 159.68 | 0.00 | 10994.36 |
| 2007 | 31.88 | 51.48 | 5.53 | 5246.138 |
| 2008 | 56.55 | 106.39 | 0.19 | 9288.462 |
| 2009 | 54.96 | 134.51 | 0.00 | 9566.501 |
| 2010 | 28.06 | 69.32 | 0.00 | 4550.792 |
| 2011 | 52.15 | 97.09 | 3.43 | 8572.652 |
| 2012 | 25.30 | 61.24 | 0.19 | 4576.585 |
| 2013 | 41.00 | 96.38 | 0.00 | 7010.279 |
| 2014 | 23.48 | 49.71 | 0.00 | 4271.341 |

**Table S2**. Mean, maximal, minimal and cumulated (CND2) questing nymph densities of the second semester of each year, given in number of nymphs per 100m^2^. For this latter measure only (CND2), fictive null samplings were considered (see Tick sampling part in Material and Methods for details).

| **Year** | **Mean density** | **Maximal density** | **Minimal density** | **CND2** |
| --- | --- | --- | --- | --- |
| 2000 | 18.44 | 39.08 | 2.00 | 3106.006 |
| 2001 | 23.26 | 37.75 | 0.09 | 3821.434 |
| 2002 | 12.34 | 21.73 | 1.14 | 2409.196 |
| 2003 | 13.33 | 40.32 | 0.09 | 2190.324 |
| 2004 | 6.86 | 15.92 | 1.62 | 1722.814 |
| 2005 | 19.40 | 42.04 | 4.10 | 2727.117 |
| 2006 | 10.99 | 20.40 | 0.00 | 2740.747 |
| 2007 | 6.44 | 13.73 | 0.00 | 1302.714 |
| 2008 | 14.39 | 36.32 | 0.00 | 3480.747 |
| 2009 | 5.48 | 8.77 | 0.00 | 1660.763 |
| 2010 | 9.26 | 26.12 | 0.00 | 2132.37 |
| 2011 | 9.98 | 28.41 | 0.00 | 2257.659 |
| 2012 | 11.67 | 22.38 | 1.33 | 1880.749 |
| 2013 | 3.79 | 11.14 | 0.00 | 1211.451 |
| 2014 | 10.78 | 22.85 | 1.33 | 2113.952 |
